# Defects in DNA double‐strand break repair resensitize antibiotic‐resistant *Escherichia coli* to multiple bactericidal antibiotics

**DOI:** 10.1002/mbo3.1316

**Published:** 2022-09-23

**Authors:** Sarah A. Revitt‐Mills, Elizabeth K. Wright, Madaline Vereker, Callum O'Flaherty, Fairley McPherson, Catherine Dawson, Antoine M. van Oijen, Andrew Robinson

**Affiliations:** ^1^ School of Chemistry and Molecular Bioscience, Molecular Horizons Institute University of Wollongong Wollongong New South Wales Australia; ^2^ Illawarra Health and Medical Research Institute Wollongong New South Wales Australia

**Keywords:** AMR, antimicrobial resistance, DNA repair, double‐strand break repair, resensitization, SOS response

## Abstract

Antibiotic resistance is becoming increasingly prevalent amongst bacterial pathogens and there is an urgent need to develop new types of antibiotics with novel modes of action. One promising strategy is to develop resistance‐breaker compounds, which inhibit resistance mechanisms and thus resensitize bacteria to existing antibiotics. In the current study, we identify bacterial DNA double‐strand break repair as a promising target for the development of resistance‐breaking co‐therapies. We examined genetic variants of *Escherichia coli* that combined antibiotic‐resistance determinants with DNA repair defects. We observed that defects in the double‐strand break repair pathway led to significant resensitization toward five bactericidal antibiotics representing different functional classes. Effects ranged from partial to full resensitization. For ciprofloxacin and nitrofurantoin, sensitization manifested as a reduction in the minimum inhibitory concentration. For kanamycin and trimethoprim, sensitivity manifested through increased rates of killing at high antibiotic concentrations. For ampicillin, repair defects dramatically reduced antibiotic tolerance. Ciprofloxacin, nitrofurantoin, and trimethoprim induce the promutagenic SOS response. Disruption of double‐strand break repair strongly dampened the induction of SOS by these antibiotics. Our findings suggest that if break‐repair inhibitors can be developed they could resensitize antibiotic‐resistant bacteria to multiple classes of existing antibiotics and may suppress the development of de novo antibiotic‐resistance mutations.

## Graphical abstract

Antimicrobial resistance (AMR) poses a significant global health threat; there is an urgent need to develop new types of therapeutics with novel modes of action. This study identified bacterial DNA double‐strand break repair as a promising target for the development of resistance‐breaking co‐therapies. We observed that defects in the double‐strand break repair pathway led to significant resensitization of antibiotic‐resistant *Escherichia coli* toward five bactericidal antibiotics representing different functional classes.

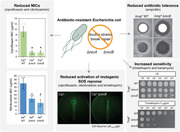

## 1 INTRODUCTION

The emergence of antimicrobial resistance (AMR) poses a significant global health threat, with once trivial bacterial infections becoming increasingly difficult to treat (Bush et al., 2011). AMR has rendered several current antibiotics effectively obsolete, severely limiting infection treatment options (Levy & Marshall, 2004; Ventola, 2015). There is significant interest in developing combinational drugs that can extend the clinical lifetimes of current therapeutics (Brooks & Brooks, 2014; Tamma et al., 2012). One possibility is the development of “resistance breaking” compounds that increase sensitivity to current antimicrobial therapies (Brown, 2015; Laws et al., 2019).

Many drugs and chemicals are known to induce the SOS response in bacteria, including antidepressants, antivirals, herbicides, and anticancer therapies (Crane et al., 2021; Maier et al., 2018; Mamber et al., 1990). Now there is growing evidence that treatment with certain antibiotics can elevate bacterial mutation rates, potentially increasing the likelihood that antibiotic resistance mutations will appear in bacterial populations (Baharoglu & Mazel, 2011; Gutierrez et al., 2013; Kohanski, Depristo, et al., 2010; Pribis et al., 2019). For any new therapy, it would be desirable to limit the possibility of mutation by (i) narrowing the mutant selection window (Drlica & Zhao, 2007) and (ii) suppressing mutagenesis (Blázquez et al., 2018). The current study identifies bacterial DNA double‐strand break repair (DSBR) as a promising target for the development of such therapies.

Several commonly used bactericidal antibiotics have been shown to damage bacterial DNA either as a direct consequence of their primary mode of action or through secondary effects (Kohanski, Dwyer, et al., 2010). Many forms of DNA damage are lethal to bacteria if left unrepaired (Friedberg et al., 2005). Bacteria have sophisticated systems to repair DNA damage and the action of these repair pathways effectively offsets killing by DNA‐damaging antibiotics (Bjedov et al., 2003). Recent studies have demonstrated that the inactivation of bacterial DNA repair pathways can sensitize bacterial cells to multiple antibiotics. Inactivation of *recA*, a key contributor to DNA repair via homologous recombination, has been shown to reduce minimum inhibitory concentrations (MICs) against the antibiotics ceftazidime (β‐lactam; cephalosporin), fosfomycin (phosphonic antibiotic), ciprofloxacin (quinolone), trimethoprim (dihydrofolate synthesis inhibitor), and colistin (polymixin) (Thi et al., 2011). Promisingly, deletion of *recA* also resensitized a ciprofloxacin‐resistant strain of *Escherichia coli* to clinically approachable levels of ciprofloxacin (Recacha et al., 2017). The *recA* gene is required for the repair of both double‐stranded DNA breaks and single‐stranded DNA (Del Val et al., 2019). It is unclear whether these antibiotic‐sensitizing effects stem from defects in the DSBR or single‐strand gap repair (SSGR) pathways. It is also unclear whether these resensitization effects extend to other classes of bactericidal antibiotics. In this study, we aim to address these shortfalls by measuring MICs, examining time‐kill kinetics, and determining antibiotic tolerance phenotypes for *E. coli* strains defective in DSBR and SSGR. Five antibiotics documented as having bactericidal effects were examined: ciprofloxacin (Drlica et al., 2009), nitrofurantoin (McOsker & Fitzpatrick, 1994), kanamycin (Davis, 1987), trimethoprim (Giroux et al., 2017), and ampicillin (Rolinson et al., 1977).

In *E. coli*, double‐strand DNA breaks are primarily repaired through homologous recombination via the RecA protein and RecBCD pathway (Kowalczykowski et al., 1994). Single‐strand gaps are predominantly repaired by RecF, RecO, and RecR proteins through their aiding in RecA‐mediated homologous recombination (Morimatsu & Kowalczykowski, 2003). A third pathway that is utilized under DNA damage conditions is nucleotide pool sanitation (NPS). The NPS pathway removes oxidized nucleotides from the resource pool and thus prevents the insertion of aberrant bases during DNA synthesis (Fowler & Schaaper, 1997). In the absence of one particular NPS enzyme, MutT, insertion of the aberrant base 8‐oxo‐dGTP into the DNA triggers a form of maladaptive DNA repair that can kill bacterial cells (Giroux et al., 2017). We examined the effects of disrupting MutT alongside the DSBR and SSGR pathways in this study.

DNA damage also induces a mutation‐promoting stress‐response mechanism called the SOS response (Maslowska et al., 2019). In some circumstances, induction of the SOS response has been observed to increase the frequency of antibiotic‐resistance mutations that appear in bacterial populations (Blázquez et al., 2018; Cirz et al., 2005). Among the ~40 genes induced during SOS are genes that encode error‐prone DNA polymerases known to cause an array of mutations (Goodman & Woodgate, 2013). SOS is induced by RecA* nucleoprotein filaments that form in response to DNA damage (Simmons et al., 2008). Through disruption of *recA* and other DNA‐repair genes, there is potential to attenuate SOS mutagenesis. In support of this, previous work has demonstrated that *E. coli* lacking SOS‐induced genes involved in DNA repair (including *recA*) exhibit significant increases in ciprofloxacin susceptibility (Tran et al., 2016). Similar findings were again observed in genetic screening and gene expression analysis of SOS response mutant strains in intermediate‐resistant *E. coli* (Klitgaard et al., 2018). Enhancing killing and decreasing mutation supply through inhibition of bacterial DNA repair pathways represents a global approach toward the resensitization of antibiotic‐resistant bacteria. In this study, we measure the induction of the SOS response by each of the five antibiotics and examine the effects of disrupting DSBR and SSGR on SOS induction.

## 2 MATERIALS AND METHODS

### 2.1 Bacterial strains, plasmids, and culture conditions

*E. coli* strains and plasmids used in this study are listed in Tables A1 and A2, respectively. *E. coli* was cultured at 37°C in lysogeny broth (LB; supplied by BD) or LB agar (1.5% (w/v); BD). As required, media were supplemented with antibiotics (Sigma) at the following concentrations unless otherwise stated kanamycin (Kan; 25 µg/ml), ampicillin (Amp; 100 µg/ml), trimethoprim (Trp; 50 µg/ml), and spectinomycin (Spec; 50 µg/ml).

### 2.2 Molecular techniques

Plasmid DNA was extracted from *E. coli* using QIAprep Spin Miniprep kits (Qiagen) as outlined by the manufacturer. *E. coli* cells were made competent and transformed as previously described (Swords, 2003). Oligonucleotides used in this study are listed in Table A3 and were synthesized by Integrated DNA Technologies (IDT). Polymerase chain reaction (PCR) amplification was performed using Quick*Taq* (Roche) as recommended by the manufacturer.

### 2.3 Strain construction and complementation

Mutant strains were constructed in the *E. coli* K12 MG1655 background (unless otherwise stated) using λ_RED_ recombination, replacing the gene of interest with a kanamycin cassette flanked by FRT sites (Datsenko & Wanner, 2000). Construction of Δ*recA*::Kan^R^ (HH020) (Ghodke et al., 2019), Δ*recB*::Kan^R^ (EAW102) (Henrikus et al., 2020), Δ*recF*::Kan^R^ (EAW629), Δ*recO*::Kan^R^ (EAW114), and Δ*recR*::Kan^R^ (EAW669) (Henrikus et al., 2019) have been described previously. EAW999 Δ*mutT*::Kan^R^ was constructed via λ_RED_ recombination using pKD46 (Datsenko & Wanner, 2000). Where applicable, the kanamycin resistance cassette was removed from strains via FLP–FRT recombination using the plasmid pLH29 (Huang et al., 1997) to obtain the kanamycin‐sensitive derivatives: HH021 (Δ*recA*::FRT), HG356 (Δ*recB*::FRT), MV009 (Δ*recF*::FRT), SRM019 (Δ*recO*::FRT), SRM020 (Δ*recR*::FRT) and MV005 (Δ*mutT*::FRT). All mutations were confirmed by PCR.

Mutant DNA repair alleles were moved into the ciprofloxacin‐resistant (Cip^R^) background (CH5741) using P1 transduction. P1 phage lysates were raised using HH020 (Δ*recA*::Kan^R^), EAW102 (Δ*recB*::Kan^R^), EAW629 (Δ*recF*::Kan^R^), EAW114 (Δ*recO*::Kan^R^) and EAW669 (Δ*recR*::Kan^R^), and EAW999 (Δ*mutT*::Kan^R^).

Kanamycin resistant (Kan^R^) strains were constructed by introducing the pUA66 plasmid (which confers kanamycin resistance through the *aph(3′)‐*II gene) into the strains MG1655 (wild‐type [WT]), HH021 (Δ*recA*::FRT), HG356 (Δ*recB*::FRT), MV009 (Δ*recF*::FRT), SRM019 (Δ*recO*::FRT), SRM020 (Δ*recR*::FRT) and MV005 (Δ*mutT*::FRT) via transformation.

Ampicillin resistant (Amp^R^) strains were constructed by introducing the pWSK29 plasmid (which confers ampicillin resistance through the *bla* gene) into the strains MG1655 (WT), HH020 (Δ*recA*::Kan^R^), EAW102 (Δ*recB*::Kan^R^), EAW629 (Δ*recF*::Kan^R^), EAW114 (Δ*recO*::Kan^R^). EAW669 (Δ*recR*::Kan^R^) and EAW999 (Δ*mutT*:: Kan^R^) via transformation.

Trimethoprim resistant (Tmp^R^) strains were constructed through λ RED recombination of SRP84 (Table A3) with MG1655. This recombination introduced a single point mutation from C>T in position 49765 of the chromosome (EKW048). The mutation was confirmed by PCR amplification and sequencing. P1 phage lysates were then raised using HH020 (Δ*recA*::Kan^R^), EAW102 (Δ*recB*::Kan^R^), EAW629 (Δ*recF*::Kan^R^), EAW114 (Δ*recO*::Kan^R^), and EAW669 (Δ*recR*::Kan^R^), EAW999 (Δ*mutT*::Kan^R^), and transduced with EKW048.

Nitrofurantoin resistant (Nit^R^) strains were constructed using P1 transduction. P1 phage lysate were raised on JW0835‐1 (*nfsA*::kan^R^) from the Keio collection (Baba et al., 2006) and transduced into the strains MG1655 (WT), HH021 (Δ*recA*::FRT), HG356 (Δ*recB*::FRT), MV009 (Δ*recF*::FRT), SRM019 (Δ*recO*::FRT), SRM020 (Δ*recR*::FRT) and MV005 (Δ*mutT*::FRT). Kanamycin resistance was cured after each transduction and subsequently cured of the pLH29 plasmid to produce antibiotic‐sensitive variants.

For complementation of the Δ*recA* mutants, the plasmid, pHG134 (also referred to as pRecA), was used as described previously (Ghodke et al., 2019). For the complementation of Δ*recB* mutants, the plasmid pSRM3 (referred to as pRecB) was constructed by Aldevron (GenBank accession number: OP341514). The *recB* gene and 200 bp upstream were synthesized and cloned into KpnI/XbaI restriction sites on the pJM1071 plasmid backbone. Complementation plasmids were introduced into the appropriate strains by transformation.

### 2.4 MIC strip assays and TD tests

MICs were primarily determined using Liofilchem® MTS™ (MIC Test Strips) according to the manufacturer's recommendations. When necessary, agar plates were supplemented with zinc pyrithione (ZnPT) at concentrations of 1, 3, 10, 30, 100 μM, or iron(III) phthalocyanine‐4,4′,4″,4‴‐tetrasulfonic acid (FePcTs) at a concentration of 25 µM. When required, TD tests (Gefen et al., 2017) were performed following the MIC strip assay. The antibiotic strip was removed from the plate and 5 µl of 40% (w/v) D‐glucose solution was then added and left to dry at room temperature. Plates were further incubated overnight at 37°C. Tolerance was described as the growth of colonies in the zone of inhibition (ZOI) following the addition of glucose.

### 2.5 Disc diffusion assays and TD tests

Where MIC test strips were unavailable or strain MICs were beyond the strip test range, disc diffusion assays were performed. Cells were grown in 500 µl LB broth for roughly 6 h at 37°C. Then, 100 µl of culture was plated onto an LB agar plate. A sterile 13 mm Whatman® disc (GE Healthcare) with antibiotic (or compound) was placed in the center of the plate. Final antibiotic/compound concentrations on the discs were as follows unless otherwise stated: 10 mg ampicillin, 1 mg trimethoprim, 3.5 mg kanamycin, 50 µg ML328, 15 µg IMP‐1700. Plates were incubated at 37°C overnight. The ZOI surrounding the disc was measured. When required, a modified TD test (Gefen et al., 2017) was performed following the disc diffusion assay as described above.

### 2.6 Tolerance regrowth percentage and ZOI area calculations

Regrowth percentages and the area within the ZOI were measured using ImageJ (Schneider et al., 2012). Areas, and subsequent regrowth, under the MIC strip or antibiotic disc, were excluded from these measurements. Images of MIC plates were imported, cropped, and aligned using regular ImageJ functions. To select the ZOI, images were subject to thresholding using the “auto‐threshold” mean preset function. The ZOI was selected using the “wand” tool and added to the ROI manager. The scale was set to pixels and the area was measured using the “measurements function.”

For TD image processing, ZOI selection and measurements were conducted as above. To select any colonies within the ZOI, all thresholded regions were selected using the “select all” function and added to the ROI manager. Any bacterial growth already present within the ZOI was included in measurements as follows. The area within the ZOI without bacterial growth was determined by selecting the two ROIs and using the “AND” and measurement functions. TD images were cropped and aligned as above. TD images were subject to thresholding using the “auto‐threshold” mean preset function. The ZOI from the MIC plates was added to the TD image and the position was adjusted as needed. Colonies were selected as before using the “select all” function. The area within the ZOI without colonies was measured using the “AND” and measurement functions. Measurements were exported and further processed in excel. Percent regrowth was calculated using the following equation:

$$\%\;\mathrm{regrowth}=\frac{\left(\mathrm{TD}\;\mathrm{area}\;\mathrm{without}\;\mathrm{colonies}‐\mathrm{MIC}\;\mathrm{area}\;\mathrm{without}\;\mathrm{colonies}\right)}{\mathrm{Zone}\;\mathrm{of}\;\mathrm{inhibition}\;\mathrm{area}}\times 100.$$

Any area or regrowth under antibiotic strips or discs was not included in the regrowth measurements. The ImageJ macro code used to analyze images is available in Appendix 3.

### 2.7 Time‐kill experiments

To determine the viability of wild‐type and mutant strains, viability was examined using time‐kill assays. Single colonies were used to inoculate 1 ml Mueller‐Hinton (MH) cation adjusted broth and incubated 850 rpm at 37°C overnight. The overnight culture was reset at 1:100 in 10 ml MH broth and incubated shaking at 200 rpm until an OD_600_ of 0.4 was reached. Where needed, OD_600_ values were normalized to 0.4. A 2 ml sample of culture was then incubated with 3 µg/ml (3× MIC), 5 µg/ml (5× MIC), or 10 µg/ml (10× MIC) kanamycin for 3 h. Samples (200 µl) were taken hourly and washed three times in 0.1 M MgSO_4_. Serial dilutions were prepared in LB to 10^−6^ and 5 µl of each dilution was spotted in duplicate onto MH agar plates. Plates were dried and incubated at 37°C overnight. Colonies were enumerated and cfu/ml was determined for each time point.

### 2.8 Antibiotic sensitivity assays (spot‐plate assays)

Single colonies were used to inoculate 1 ml LB broth and incubated at 850 rpm at 37°C overnight. The following day, the overnight culture was reset at 1:100 in LB broth and incubated shaking until an OD_600_ of 0.2 was reached. Serial dilutions were prepared to 10^−5^ and 5 µl of each dilution was spotted in duplicate onto LB agar plates and LB agar with an antibiotic. Antibiotics were added to final concentrations of 650 and 775 µg/ml kanamycin, 0.2 µg/ml trimethoprim (sensitive), and 5 µg/ml trimethoprim (Tmp^R^). Plates were allowed to dry and incubated at 37°C overnight. Plates were imaged using a BioRad GelDoc imager.

### 2.9 MIC/minimum bactericidal concentration broth assays

Where necessary, MICs were determined using a 96‐well plate following the EUCAST broth microdilution protocol. Single colonies were used to inoculate 1 ml MH cation adjusted broth and incubated at 37°C overnight. The overnight culture was diluted at 1:100 in MH broth and incubated shaking at 850 rpm for 3 h. Exponential phase cells were then diluted at 1:200. Ten microliters of culture (approximately 10^5^ cfu/ml) were used to inoculate wells containing 190 µl MH broth supplemented with dimethyl sulfoxide (DMSO)/compound, ensuring a 2% (v/v) DMSO final concentration in all wells. Compounds were prepared in twofold serial dilutions. Plates were incubated at 37°C in a PolarStar Omega plate reader with shaking. The optical density at 600 nm (OD_600_) was recorded every 20 min for 18 h. OD_600_ measurements were background corrected against no‐inoculum controls. The MIC was defined as the lowest concentration of compound with no growth as determined by OD_600_ readings. MIC values were calculated using data from at least three biological replicates. IC_50_ values were determined by dose–response nonlinear regression.

### 2.10 SOS plate assays

SOS induction in response to antibiotic exposure was analyzed qualitatively by an agar plate‐based fluorescence assay. This assay used strains containing a reporter plasmid that expresses the fast folding GFP derivative, GFPmut2 (Zaslaver et al., 2006), under the control of the SOS‐inducible promoter P_*sulA*_. Cells were grown in 500 µl LB broth supplemented with kanamycin (to select for the reporter plasmid) for roughly 6 h at 37°C. Then, 100 µl of culture was used to inoculate 4 ml of soft LB agar (0.5% agar (w/v)). The agar was then poured on top of a regular LB Kan plate (to maintain selection for the reporter plasmid) and allowed to set. A Liofilchem® MTS™ (MIC Test Strips), or sterile 13 mm Whatman® disc (GE Healthcare) with antibiotic was placed in the center of the plate. Plates were incubated at 37°C for 24 h. The fluorescence signal was detected using custom‐built fluorescence photography set up using an Andor Zyla 5.5 camera equipped with a Nikon 18–55 mm SLR objective and a Chroma ZET405/488/594m‐TRF emission filter. Excitation was achieved with a Thorlabs DC4104 high‐power LED source behind a Chroma ZET405/488/594x excitation filter. GFP was visualized using 490 nm wavelength excitation at a power of 350 mW. Exposure times were adjusted as needed according to signal strength. The camera and LED light source were controlled using MicroManager software (Edelstein et al., 2014).

## 3 RESULTS

### 3.1 Disrupting DSBR resensitizes ciprofloxacin‐resistant and nitrofurantoin‐resistant *E. coli*

It is widely accepted that ciprofloxacin and other fluoroquinolones induce DNA damage in bacteria (Drlica et al., 2009; McOsker & Fitzpatrick, 1994). Ciprofloxacin targets the essential bacterial enzymes DNA gyrase and topoisomerase IV, stabilizing a protein‐bridged DNA double‐strand break intermediate that is formed during supercoiling and decatenation reactions (Drlica et al., 2008, 2009; Henrikus et al., 2020; Hong et al., 2019). Cells treated with ciprofloxacin are known to accumulate DNA double‐strand breaks (Drlica et al., 2009; Henrikus et al., 2020). The mechanism(s) underlying the formation of ciprofloxacin‐induced breaks remain under investigation (Drlica et al., 2008; Wang & Zhao, 2009). Resistance to ciprofloxacin commonly develops through the acquisition of mutations in the genes encoding DNA gyrase and topoisomerase IV. Ciprofloxacin has a reduced affinity for these mutant forms of DNA gyrase and topoisomerase IV (Heisig, 1996; Yoshida et al., 1990). In this study, we made use of a ciprofloxacin‐resistant (Cip^R^) derivative of *E. coli*, CH5741 (Huseby et al., 2017). This strain has clinically relevant point mutations in the genes of the quinolone targets: DNA gyrase (*gyrA;* [S83L, D87N]) and topoisomerase IV (*parC;* [S80I]). The introduction of these point mutations increases ciprofloxacin MIC 1000‐fold in comparison to the sensitive background (Huseby et al., 2017). It is assumed that double‐strand breaks are still formed in this background, but due to reduced target affinity, far higher concentrations of ciprofloxacin would be required.

**Figure 1:**
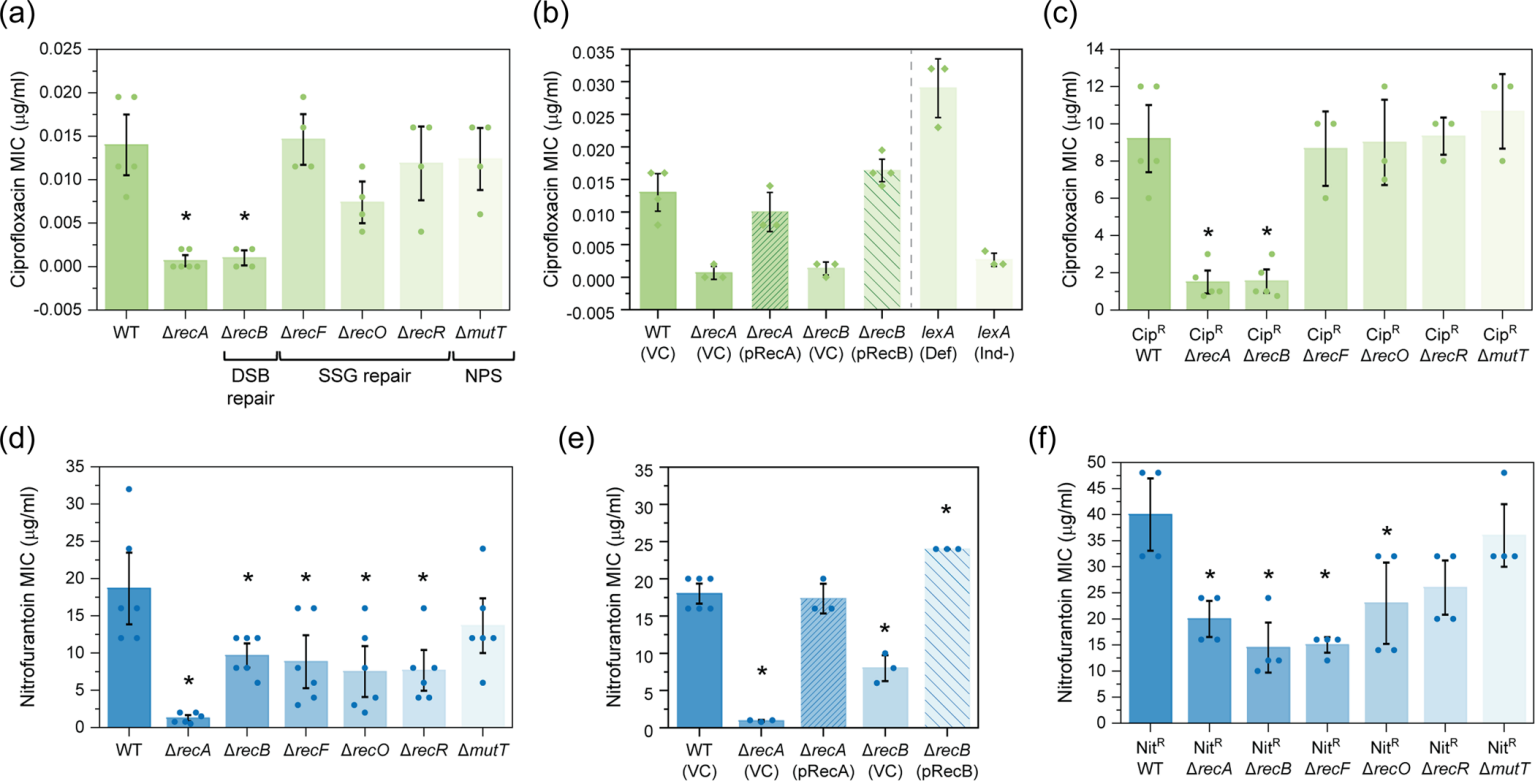
Cells deficient in double‐strand break repair have an increased sensitivity to ciprofloxacin and nitrofurantoin. (a) Ciprofloxacin minimum inhibitory concentration (MIC) values obtained for isogenic *Escherichia coli* strains MG1655 (WT; wild‐type), Δ*recA*::Kan^R^ (HH020), Δ*recB*::Kan^R^ (EAW102), Δ*recF*::Kan^R^ (EAW629), Δ*recO*::Kan^R^ (EAW114), Δ*recR*::Kan^R^ (EAW669), and Δ*mutT*::Kan^R^ (EAW999). The means and standard errors of the mean are shown, based on results from at least four biological replicates. Statistical analysis was carried out using two‐sample Student's *t*‐tests. An asterisk denotes statistical significance (*p* < 0.05) compared to wild‐type (MG1655). (b) Ciprofloxacin MIC values obtained for the *E. coli* strains wild‐type (MG1655) with empty vector (VC; vector control), Δ*recA* and Δ*recB* mutants with empty vector and complemented derivatives (pRecA and pRecB, respectively), *lexA* defective (*lexA*(Def))and *lexA* induction defective (*lexA*(Ind‐). The means and standard errors of the mean are shown based on results from at least three biological replicates. Statistical analysis was carried out using two‐sample Student's *t*‐tests. An asterisk denotes statistical significance (*p* < 0.05) compared to wild‐type with empty vector, WT (VC). (c) Ciprofloxacin MIC values obtained for isogenic ciprofloxacin‐resistant (Cip^R^) DNA repair‐deficient *E. coli* strains Cip^R^ (CH5741), Cip^R^ Δ*recA* (FM002), Cip^R^ Δ*recB* (FM001), Cip^R^ Δ*recF* (FM003), Cip^R^ Δ*recO* (FM004), Cip^R^ Δ*recR* (FM005), and Cip^R^ Δ*mutT* (MV001). The means and standard errors of the mean are shown, based on results from at least three biological replicates. Statistical analysis was carried out using two‐sample Student's *t*‐tests. An asterisk denotes statistical significance (*p* < 0.05) compared to Cip^R^. (d) Nitrofurantoin MIC values obtained for isogenic *E. coli* strains. The means and standard errors of the mean are shown, based on results from at least six biological replicates. Statistical analysis was carried out using Student's *t*‐tests. An asterisk denotes sta*t*istical significance (*p* < 0.05) compared to wild‐type (MG1655). (e) Nitrofurantoin MIC values obtained for the *E. coli* strain wild‐type (MG1655) with empty vector (VC; vector control), Δ*recA* and Δ*recB* mutants with empty vector, and complemented derivatives (pRecA and pRecB, respectively). The means and standard errors of the mean are shown based on results from at least three biological replicates. Statistical analysis was carried out using Student's *t*‐tests. An asterisk denotes statistical significance (*p* < 0.05) compared to wild‐type with empty vector, WT (VC). (f) Nitrofurantoin MIC values obtained for isogenic nitrofurantoin‐resistant (Nit^R^) DNA repair‐deficient *E. coli* strains Nit^R^ (EKW046), Nit^R^ Δ*recA* (EKW047), Nit^R^ Δ*recB* (EKW048), Nit^R^ Δ*recF* (EKW049), Nit^R^ Δ*recO* (EKW050), Nit^R^ Δ*recR* (EKW051) and Nit^R^ Δ*mutT* (EKW052). Statistical analysis was carried out using Student's *t*‐tests. The means and standard errors of the mean are shown, based on results from at least three biological replicates. An asterisk denotes statistical significance (*p* < 0.05) compared to Nit^R^.

Reasoning that bacterial DNA repair might offset the killing effects of ciprofloxacin in both sensitive and resistant backgrounds, we examined the sensitivities of *E. coli* strains that combined defects in the DSBR, SSGR, and NPS repair pathways with ciprofloxacin‐resistance mutations. We determined the MIC for each strain using Liofilchem® MTS™ (MIC Test Strips). Cells deficient in RecA or RecB were hypersensitive to ciprofloxacin in comparison to the wild‐type (ciprofloxacin‐sensitive) background (Figure 1a). This phenotype was rescued upon complementation with *recA* and *recB* in trans (Figure 1b), confirming the involvement of both RecA and the DSBR pathway in ciprofloxacin sensitivity. Cells lacking the SSGR protein RecO initially appeared to be more sensitive to ciprofloxacin than wild‐type cells, but this was found not to be statistically significant. Deletion of other genes whose products are involved in single‐stranded DNA repair (*recF* and *recR)* or nucleotide sanitation (*mutT*) had no significant effect on ciprofloxacin MIC. These findings indicate that DSBR is required for the repair of DNA damage following exposure to ciprofloxacin, in agreement with previous studies (Dörr et al., 2009; Henrikus et al., 2020).

To determine if these findings translated to an antibiotic‐resistant background, the sensitization effect of disrupting DNA repair genes was examined using a ciprofloxacin‐resistant (Cip^R^) derivative of *E. coli*, CH5741 (Huseby et al., 2017). Deletion of *recA* and *recB* led to significant resensitization toward ciprofloxacin, reducing the respective MICs sevenfold and sixfold in comparison to the ciprofloxacin‐resistant parental strain (Figure 1c). Disruption of SSGR and NPS did not alter MIC in the Cip^R^ background. These results support our assumption that double‐strand breaks are formed in the Cip^R^ background and indicate that disruption of DSBR reduces resistance in *E. coli* that have already acquired Cip^R^ mutations.

Furthermore, the role of LexA as a repressor of the SOS response (via the production of RecA*) and the effect of this response on antibiotic sensitivity was tested. The ciprofloxacin MIC assays were repeated for strains containing mutations in *lexA*. Cells with a defective LexA (*lexA*[Def]) (Robinson et al., 2015) exhibit a constitutive SOS response. These cells demonstrated an elevated MIC, indicating that constitutive SOS may be protective. Conversely, *E. coli* mutants containing an induction‐deficient LexA (*lexA*[Ind‐]) (Ennis et al., 1985; Henrikus et al., 2020), whereby the SOS response could not be activated, resulted in highly sensitized cells with a MIC of <0.002 μg/ml, similar to Δ*recA* mutants. This study suggests that the SOS response is an important factor in antibiotic resistance, and without induction of the SOS response cells are severely sensitized to ciprofloxacin.

Nitrofurantoin has previously gone through a period of decreased use due to fears of toxic side effects, yet it has re‐emerged as an ISDA‐endorsed first line‐drug in the treatment of urinary tract infections (UTI; Dason et al., 2011). While this drug is commonly used now, it still shows a low propensity for resistance (Gardiner et al., 2019). The mechanism of action for nitrofurantoin is poorly understood. Studies suggest two mechanisms: (i) inhibition of ribosomes, and consequently, protein synthesis (McOsker & Fitzpatrick, 1994); (ii) direct damage to DNA (Jenkins & Bennett, 1976). Nitrofurantoin is a prodrug. Conversion from the prodrug to active drug form requires nitrofurantoin to be processed intracellularly by the bacterial nitroreductases NfsA and NfsB (Bryant et al., 1981). Resistance to nitrofurantoin is associated with loss‐of‐function mutations in these two nitroreductases, which results in the drug remaining in the inactive prodrug state (McCalla et al., 1978). In the current study, nitrofurantoin‐resistant (Nit^R^) strains were constructed through the deletion of *nfsA*, which encodes the nitroreductase NfsA. At the outset of the study, it was not known whether this mutation would eliminate nitrofurantoin‐induced DNA damage or not.

In the nitrofurantoin‐sensitive background, both Δ*recA* and Δ*recB* cells were found to be hypersensitive to nitrofurantoin (Figure 1d). Deletion of *recA* resulted in a 15× reduction in MIC from 18.7 ± 2.9 μg/ml (wild‐type) to 1.25 ± 0.3 μg/ml. Cells deficient in DSBR (Δ*recB*) demonstrated a MIC half that of the wild‐type strain (9.7 ± 0.8 μg/ml). This effect was then rescued upon complementation (Figure 1e). The deletion of the genes *recF*, *recO*, or *recR*, which encode SSGR proteins, also significantly reduced MIC compared to wild‐type. Deletion of nucleotide sanitation (Δ*mutT*) did not affect MIC. In the nitrofurantoin resistant (Nit^R^) background, deletion of *recA, recB*, or *recF* led to significant resensitization (Figure 1f). Deletion of *recA* fully resensitized Nit^R^ cells to the wild‐type level (20 ± 2.6 μg/ml). Deletion of *recB* or *recF* was even more sensitizing, reducing the MIC to below wild‐type levels (14.5 ± 3.7 and 15± 1.2 μg/ml, respectively). Cells lacking other SSGR proteins, RecO and RecR, in addition to MutT showed no significant resensitization. The potent resensitization effects of *recA*, *recB*, and *recF* mutations strongly suggest that DNA damage still occurs in cells that have developed nitrofurantoin resistance through loss of function mutations in NfsA. Disruption of the DSBR pathway fully resensitizes NfsA‐lacking cell activity toward nitrofurantoin.

### 3.2 Disrupting DSBR enhances killing by kanamycin and trimethoprim

The antibiotics kanamycin and trimethoprim target essential components of the bacterial cell, namely ribosomes and folate biosynthesis (Kohanski, Dwyer, et al., 2010; Visentin et al., 2012). While the primary action of these antibiotics does not directly induce DNA damage, there is now growing evidence that treatment of bacterial cells with bactericidal antibiotics results in the overproduction of reactive oxygen species (ROS) (Dwyer et al., 2014). It has been suggested that treatment with these antibiotics provokes the accumulation of ROS leading to DNA damage (Belenky et al., 2015; Dwyer et al., 2014; Foti et al., 2012; Wang & Zhao, 2009). We, therefore, examined whether cells lacking DSBR, SSGR, and NPS are sensitized to kanamycin and trimethoprim.

**Figure 2:**
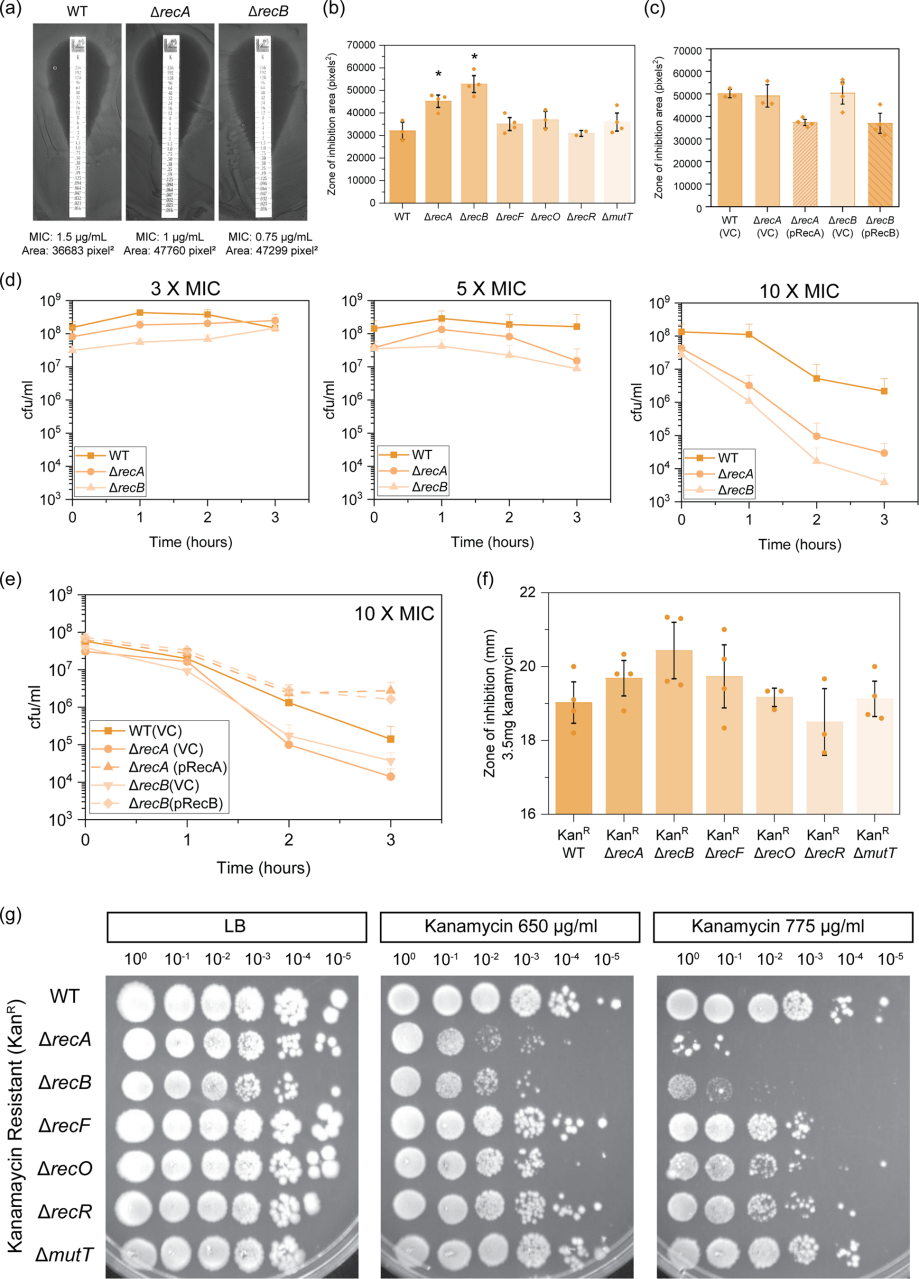
Deletion of double‐strand break repair results in moderate sensitivity to the aminoglycoside kanamycin. (a) Representative images of kanamycin MIC plate assays for wild‐type (WT), Δ*recA*, and Δ*recB Escherichia coli* strains. MICs and measured zone of inhibition areas in pixels^2^ are denoted below the images. (b) Zone of inhibition (ZOI) area measurements for isogenic *E. coli* strains MG1655 (WT; wild‐type), Δ*recA*::FRT (HH021), Δ*recB*::FRT (HG356), Δ*recF*::FRT (MV009), Δ*recO*::FRT (SRM019), Δ*recR*::FRT (SRM020) and Δ*mutT*::FRT (MV005). ZOI areas surrounding MIC strips were measured using ImageJ (Schneider et al., 2012). The means and standard errors of the mean are shown based on results from at least three biological replicates. Statistical analysis was carried out using a Student's *t*‐test. An asterisk denotes statistical significance (*p* < 0.05) compared to wild‐type (WT; MG1655). (c) Kanamycin ZOI area values obtained for wild‐type (MG1655) with empty vector (VC; vector control), Δ*recA* and Δ*recB* mutants with empty vector, and complemented derivatives (pRecA and pRecB, respectively). The means and standard errors of the mean are shown based on results from at least three biological replicates. (d) Time‐kill survival assays for isogenic *E. coli* strain MG1655 (WT; wild‐type), Δ*recA*::FRT, Δ*recB*::FRT. Cell viability (cfu/ml) was measured hourly for 3 h following treatment with 3× MIC (3 µg/ml), 5× MIC (5 µg/ml), or 10× MIC (10 µg/ml) kanamycin. The means and standard errors of the mean are shown based on results from at least three biological replicates. (e) Complemented time‐kill survival assay (10× MIC) for wild‐type (MG1655) with empty vector (VC; vector control), Δ*recA* and Δ*recB* mutants with empty vector, and complemented derivatives (pRecA and pRecB, respectively). The means and standard errors of the mean are shown based on results from at least three biological replicates. (f) Zone of inhibition area measurements for kanamycin‐resistant WT and DNA repair mutant strains following disk diffusion assays with 3.5 mg kanamycin. Kan^R^ (CD001), Kan^R^ Δ*recA* (CD002), Kan^R^ Δ*recB* (CD003), Kan^R^ Δ*recF* (CD004), Kan^R^ Δ*recO* (S^R^M026), Kan^R^ Δ*recR* (S^R^M027) and Kan^R^ Δ*mutT* (CD005). The means and standard errors of the mean are shown based on results from at least three biological replicates. (g) Spot plate dilution assays of kanamycin‐resistant DNA repair‐deficient strains. Normalized exponential phase cells (OD_600_ 0.2) were diluted to 10^−5^ and spotted onto lysogeny broth (LB) and LB agar supplemented with 650 or 775 µg/ml kanamycin. Images show representative plates from independent triplicate replicates.

**Figure A1:**
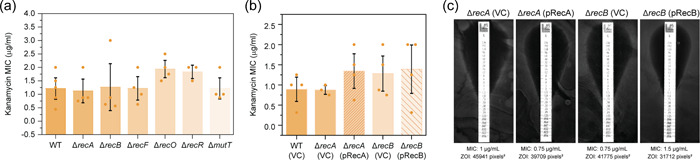
(a) Kanamycin minimum inhibitory concentration (MIC) values obtained for isogenic *Escherichia coli* strains MG1655 (WT; wild‐type), Δ*recA*::Kan^R^ (HH020), Δ*recB*::Kan^R^ (EAW102), Δ*recF*::Kan^R^ (EAW629), Δ*recO*::Kan^R^ (EAW114), Δ*recR*::Kan^R^ (EAW669) and Δ*mutT*::Kan^R^ (EAW999). MICs were assayed using MIC test strips according to the manufacturer's instructions. The means and standard errors of the mean are shown, based on results from at least four biological replicates. (b) Kanamycin MIC values obtained for wild‐type (MG1655) with empty vector (VC; vector control), Δ*recA* and Δ*recB* mutants with empty vector, and complemented derivatives (pRecA and pRecB, respectively). The means and standard errors of the mean are shown based on results from at least three biological replicates. (c) Representative images of kanamycin MIC plate assays for Δ*recA* (VC), Δ*recA* (pRecA), Δ*recB* (VC), and Δ*recB* (pRecB) *E. coli* strains. MICs and measured zone of inhibition areas in pixels^2^ are denoted below the corresponding images.

We first examined if DNA repair‐deficient strains had altered sensitivity to kanamycin by MIC tests. For most strains, MICs were similar to wild‐type (Figure A1a). Disruption of DSBR, SSGR, or NPS did not lead to reduced MICs; MICs were marginally increased in Δ*recO* (1.9 ± 0.2 µg/ml) and Δ*recR* (1.8 ± 0.2 µg/ml) mutants compared to wild‐type (1.2 ± 0.3 µg/ml). We did notice, however, that *recA* and *recB* mutants had significantly larger zones of clearing surrounding the MIC strip (Figure 2). Complementation of the *recA* and *recB* mutants did not alter MIC (Figure A1b) but did reduce the area within the ZOI (Figures 2c and A1c). We hypothesized that the enlarged ZOI might relate to improved clearance of bacterial cells at high drug concentrations. To test this idea, we used time‐kill assays to examine the killing of our *E. coli* strains following exposure to 3×, 5×, or 10× MIC kanamycin. We observed no significant killing of any strain at 3× or 5× MIC, however, at 10× MIC (Figure 2d) there was a significant killing of both Δ*recA* and Δ*recB* mutants. Complementation alleviated this sensitivity (Figure 2e). The increased sensitivity of the *recA* and *recB* mutants highlights the importance of RecA and DSBR in mediating survival following kanamycin treatment.

We next assessed if this increased sensitivity to kanamycin was also observed in a kanamycin‐resistant (Kan^R^) background. Kan^R^ derivatives of the DNA repair defective strains were constructed by transformation with the plasmid pUA66, which confers kanamycin resistance through the *aph(3′)‐*II gene (Zaslaver et al., 2006). Sensitivity was first assessed via disc diffusion assays with 3.5 mg of kanamycin. No changes in sensitivity to kanamycin were seen in Kan^R^ cells lacking components of SSGR or NPS. The ZOI appeared larger for Kan^R^ *recA* and *recB* mutants, however, neither was statistically significant (Figure 2f). As a more sensitive test, viability was assessed using a spot dilution assay. Both the Kan^R^ Δ*recA* and Δ*recB* mutants demonstrated increased sensitivity when plated on 650 µg/ml of kanamycin, with significantly greater sensitivity observed at 775 µg/ml (Figure 2g). No changes in viability were observed for the other strains tested. These findings confirm that both RecA and RecB are required for the survival of Kan^R^ cells at high drug concentrations.

**Figure 3:**
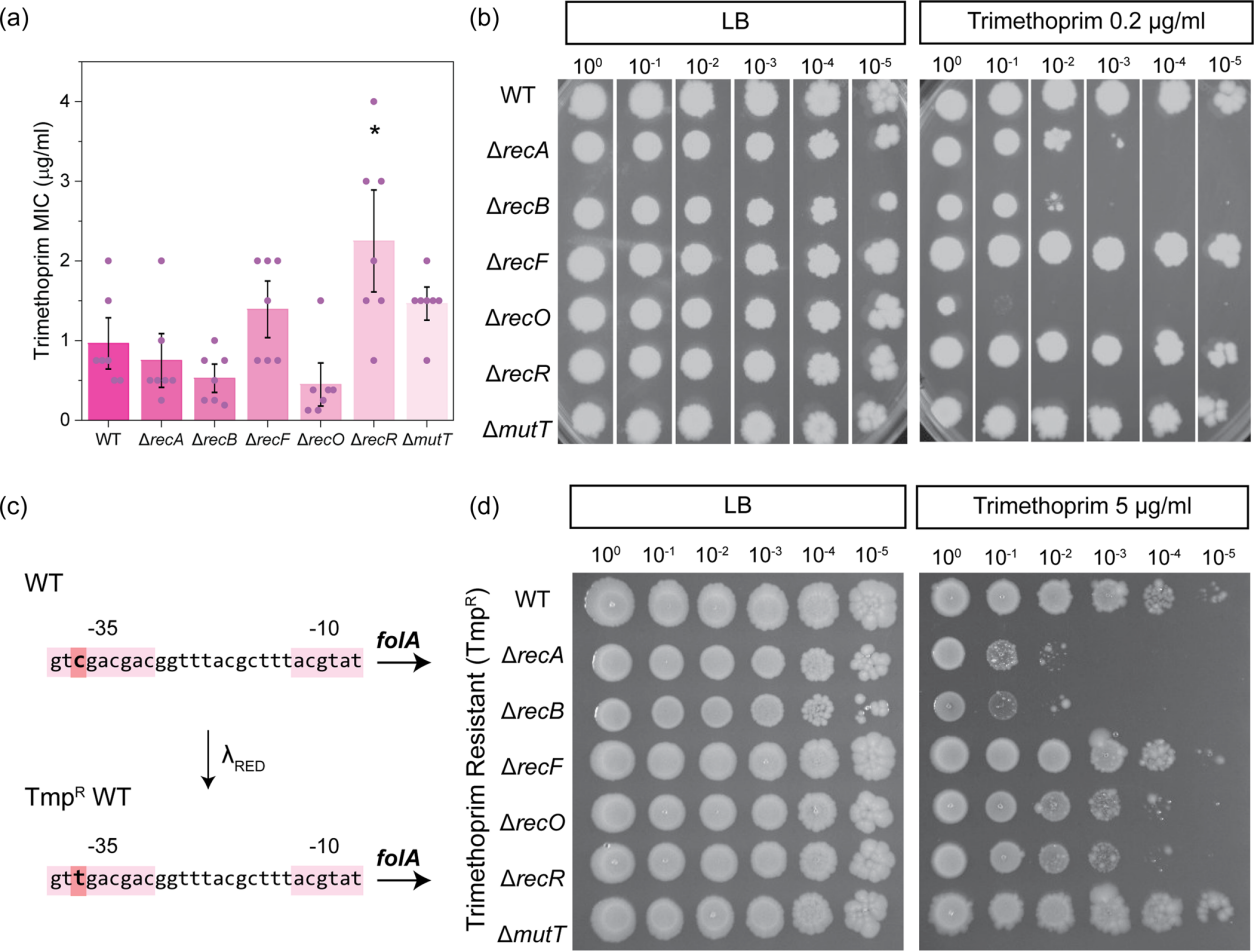
*Escherichia coli* lacking key components of DNA repair pathways demonstrate increased trimethoprim sensitivity. (a) Trimethoprim minimum inhibitory concentration values obtained for isogenic *E. coli* strains MG1655 (WT; wild‐type), Δ*recA*::Kan^R^ (HH020), Δ*recB*::Kan^R^ (EAW102), Δ*recF*::Kan^R^ (EAW629), Δ*recO*::Kan^R^ (EAW114), Δ*recR*::Kan^R^ (EAW669), and Δ*mutT*::Kan^R^ (EAW999). The means and standard errors of the mean are shown, based on results from at least four biological replicates. Statistical analysis was carried out using a Student's *t*‐test. An asterisk denotes statistical significance (*p* < 0.05) compared to wild‐type (MG1655). (b) Spot plate dilution assays of antibiotic‐sensitive DNA repair‐deficient strains (OD_600_ 0.2) spotted onto lysogeny broth (LB) and LB agar supplemented with 0.2 µg/ml trimethoprim. Images show representative plates from independent triplicate replicates. (c) Construction of the trimethoprim‐resistant *E. coli* background. The promoter region of the *folA* gene (shown as text, with the −35 and −10 regions highlighted in pink) was modified using λ_RED_ recombineering to introduce C>T single nucleotide polymorphisms (highlighted in orange) in the −35 promoter region. (d) Spot plate dilution assays of trimethoprim‐resistant DNA repair‐deficient strains, Tmp^R^ (EKW058), Tmp^R^ Δ*recA*::Kan^R^ (EKW059), Tmp^R^ Δ*recB*::Kan^R^ (EKW060), Tmp^R^ Δ*recF*:*:*Kan^R^ (EKW061), Tmp^R^ Δ*recO*:*:*Kan^R^ (EKW062), Tmp^R^ Δ*rec* ^*R*^:*:*Kan^R^ (EKW063), and Tmp^R^ Δ*mutT*::Kan^R^ (EKW064). Dilutions were spotted onto LB and LB agar supplemented with 5 µg/ml trimethoprim. Images show representative plates from independent triplicate replicates.

Trimethoprim is a bactericidal drug that disrupts folic acid biosynthesis by inhibiting the enzyme dihydrofolate reductase (DHFR) (Visentin et al., 2012). Inhibition of DHFR eventually starves the cell of nucleotides (Gleckman et al., 1981). Killing by trimethoprim in many ways mirrors the well‐studied phenomenon of thymineless death (Hong et al., 2017). As thymineless death is hypothesized to involve the formation of both double‐strand breaks and single‐strand gaps (Giroux et al., 2017; Hong et al., 2017), we examined DNA repair‐deficient strains of *E. coli* for sensitivity to trimethoprim. The Δ*recA* and Δ*recB* mutants showed no significant sensitivity in the MIC assay (Figure 3a). The SSGR mutant Δ*recO* showed sensitivity (0.27 ± 0.04 µg/ml), whereas Δ*recR* demonstrated significant resistance (2.36 ± 0.35 µg/ml) to trimethoprim treatment. We also determined cell viability using a more sensitive spot plate dilution assay (Figure 3b). Increased sensitivity to 0.2 µg/ml trimethoprim was observed for strains lacking RecA or RecB, with the greatest sensitivity observed for the RecO deficient strain. No changes in viability were seen in the other tested mutant strains when compared to wild‐type cells.

We then assessed these repair mutants in a trimethoprim‐resistant background (Tmp^R^). Resistance to trimethoprim can occur via the acquisition of mobile genetic elements or single nucleotide polymorphisms (SNPs). The most common mode of resistance in trimethoprim is SNPs within the drug's target gene *folA* which encodes for DHFR or within the *folA* promoter region (Toprak et al., 2012). For this study, trimethoprim resistance was conveyed through a clinically relevant SNP (C>T) in the *folA* −35 promoter region (Figure 3c) (Palmer et al., 2015; Toprak et al., 2012). In the Tmp^R^ background, a significant loss of cell viability at 5 µg/ml trimethoprim was observed for cells lacking RecA or RecB (Figure 3d). RecO and RecR deficient mutants showed a slight loss in viability. Thus DSBR mutations increase sensitivity in both antibiotic‐sensitive and ‐resistant backgrounds while SSGR mutants had mixed effects.

### 3.3 Defects in DSBR reduce tolerance to ampicillin

We next wanted to assess the dependency on DNA repair following treatment with an antibiotic belonging to the β‐lactam class. This family of antibiotics targets cell wall synthesis. β‐lactams block the transpeptidation of peptidoglycan subunits, reducing cell wall integrity, which increases the frequency of cell lysis events (Kohanski, Dwyer, et al., 2010). Here we chose to focus on the β‐lactam, ampicillin. Recent studies have demonstrated that treatment of *E. coli* with ampicillin increases cellular ROS levels (Dwyer et al., 2014), which are proposed to result in the damage of DNA (Belenky et al., 2015).

**Figure A2:**
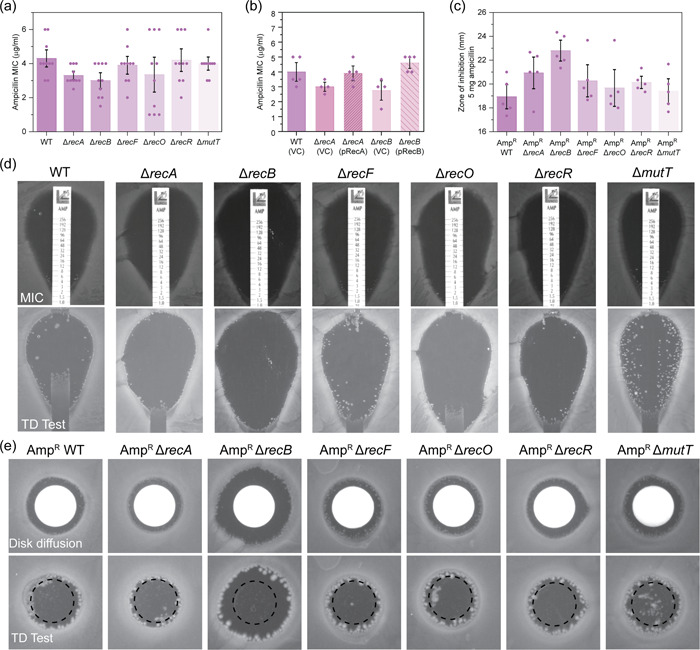
(a) Ampicillin minimum inhibitory concentration (MIC) values obtained for isogenic *Escherichia coli* strains MG1655 (WT; wild‐type), Δ*recA*::Kan^R^ (HH020), Δ*recB*::Kan^R^ (EAW102), Δ*recF*::Kan^R^ (EAW629), Δ*recO*::Kan^R^ (EAW114), Δ*recR*::Kan^R^ (EAW669) and Δ*mutT*::Kan^R^ (EAW999). MICs were assayed using MIC test strips according to the manufacturer's instructions. The means and standard errors of the mean are shown, based on results from at least three biological replicates. (b) Ampicillin MIC values obtained for the *E. coli* strains WT (MG1655) with empty vector (VC; vector control), Δ*recA* and Δ*recB* mutants with empty vector and complemented derivatives (pRecA and pRecB, respectively). The means and standard errors of the mean are shown based on results from at least three biological replicates. Statistical analysis was carried out using a Student's *t*‐test. An asterisk denotes statistical significance (*p* < 0.05) compared to wild‐type with empty vector, WT (VC). (c) Zone of inhibition area measurements for ampicillin‐resistant WT and DNA repair mutant strains following disk diffusion assays with 5 mg ampicillin. Amp^R^ (COF001), Amp^R^ Δ*recA* (COF002), Amp^R^ Δ*recB* (COF003), Amp^R^ Δ*recF* (COF004), Amp^R^ Δ*recO* (COF005), Amp^R^ Δ*recR* (COF006) and Amp^R^ Δ*mutT* (COF007). The means and standard errors of the mean are shown based on results from at least four biological replicates. (d) Representative images of ampicillin MIC and tolerance (TD Test) plate assays for isogenic *E. coli* strains MG1655 (WT; wild‐type), Δ*recA*::Kan^R^ (HH020), Δ*recB*::Kan^R^ (EAW102), Δ*recF*::Kan^R^ (EAW629), Δ*recO*::Kan^R^ (EAW114), Δ*recR*::Kan^R^ (EAW669) and Δ*mutT*::Kan^R^ (EAW999). Images show representative plates from independent triplicate replicates. (e) Representative images of ampicillin MIC and tolerance (TD Test) plate assays for isogenic *E. coli* strains Amp^R^ (COF001), Amp^R^ Δ*recA* (COF002), Amp^R^ Δ*recB* (COF003), Amp^R^ Δ*recF* (COF004), Amp^R^ Δ*recO* (COF005), Amp^R^ Δ*recR* (COF006) and Amp^R^ Δ*mutT* (COF007). Images show representative plates from independent triplicate replicates.

Following MIC analysis, we found that the sensitivity of DNA repair‐deficient *E. coli* to ampicillin was not significantly altered in comparison to wild‐type (Figure A2a). Minor differences (less than 1 µg/ml) in MIC were observed for Δ*recA* and *ΔrecB* strains, which were complemented *in trans* (Figure A2b). This change in MIC is unlikely to be clinically useful. These findings suggest that DNA repair does not play a significant role in bacterial sensitivity following ampicillin treatment.

**Figure 4:**
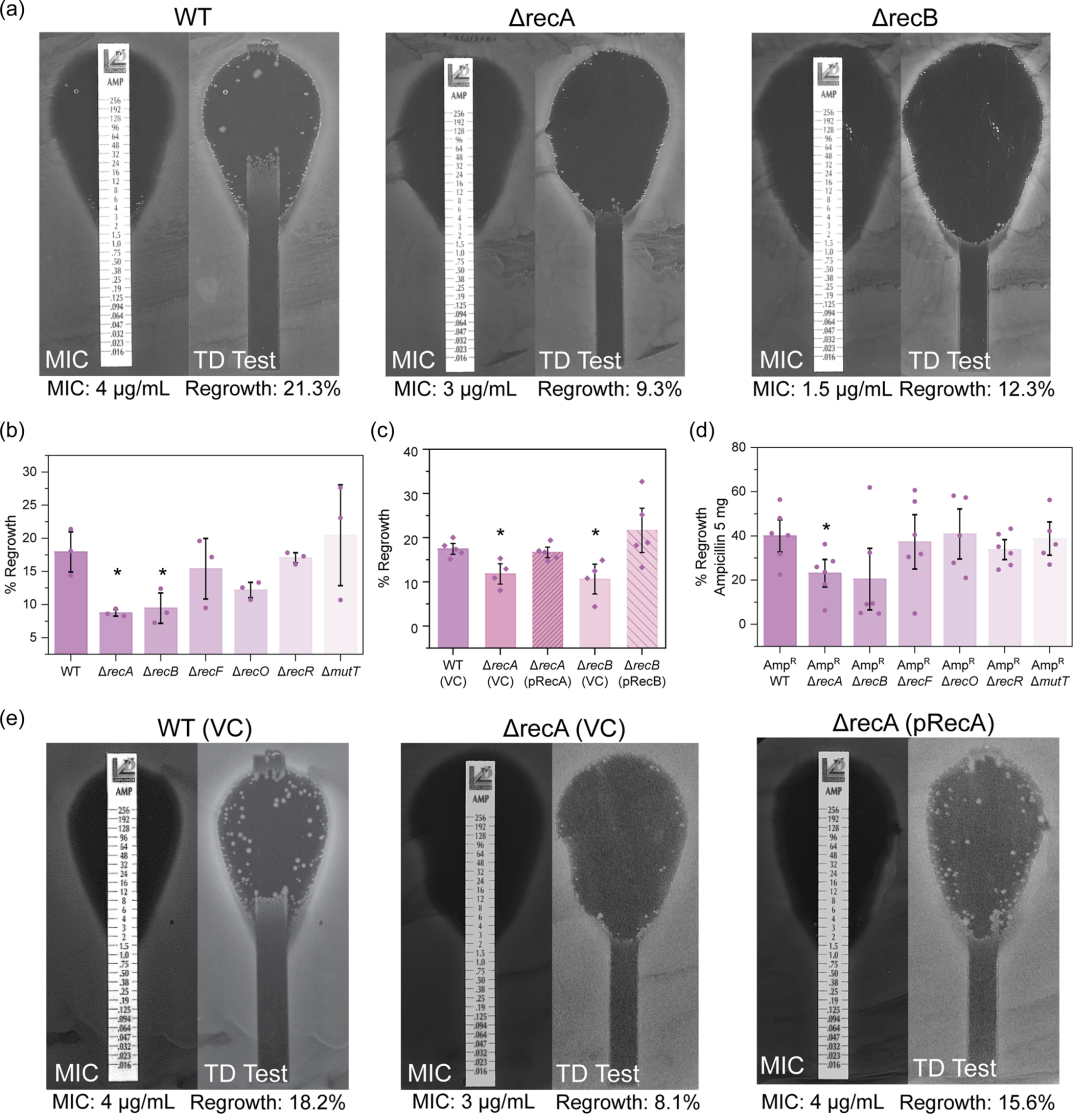
Cells deficient in double‐strand break repair have a reduced tolerance to ampicillin. (a) Representative images of ampicillin minimum inhibitory concentration (MIC) and tolerance (TD Test) plate assays for wild‐type (WT), Δ*recA*, and Δ*recB Escherichia coli* strains. MICs and percentage regrowth are denoted below the images. Images show representative plates from independent triplicate replicates. (b) Ampicillin percent regrowth values obtained for isogenic *E. coli* strains MG1655 (WT; wild‐type), Δ*recA*::Kan^R^ (HH020), Δ*recB*::Kan^R^ (EAW102), Δ*recF*::Kan^R^ (EAW629), Δ*recO*::Kan^R^ (EAW114), Δ*recR*::Kan^R^ (EAW669), and Δ*mutT*::Kan^R^ (EAW999). The means and standard errors of the mean are shown, based on results from at least three biological replicates. Statistical analysis was carried out using a Student's *t*‐test. An asterisk denotes statistical significance (*p* < 0.05) compared to wild‐type (MG1655). (c) Ampicillin percent regrowth values obtained for wild‐type (MG1655) with empty vector (VC; vector control), Δ*recA* and Δ*recB* mutants with empty vector (VC) and complemented derivatives (pRecA and pRecB, respectively). The means and standard errors of the mean are shown based on results from at least four biological replicates. Statistical analysis was carried out using a Student's *t*‐test. An asterisk denotes statistical significance (*p* < 0.05) compared to wild‐type empty vector control, WT (VC). (d) Percent regrowth measurements for ampicillin‐resistant WT and DNA repair mutant strains following disk diffusion assays with 5 mg ampicillin. Amp^R^ (COF001), Amp^R^ Δ*recA* (COF002), Amp^R^ Δ*recB* (COF003), Amp^R^ Δ*recF* (COF004), Amp^R^ Δ*recO* (COF005), Amp^R^ Δ*recR* (COF006) and Amp^R^ Δ*mutT* (COF007). The means and standard errors of the mean are shown based on results from at least five biological replicates. Statistical analysis was carried out using a Student's *t*‐test. An asterisk denotes statistical significance (*p* < 0.05) compared to the ampicillin‐resistant parental strain (Amp^R^). (e) Representative images of ampicillin MIC and tolerance (TD Test) plate assays for wild‐type with empty vector control (WT (VC)), Δ*recA* with empty vector (Δ*recA*(VC)) and complemented (Δ*recA*(pRecA)) derivatives. MICs and percentage regrowth are denoted below the images.

Ampicillin and other β‐lactam antibiotics are more effective during certain bacterial growth phases, particularly stages of high growth (Tuomanen et al., 1986). Delayed growth or dormancy can confer tolerance to ampicillin (Fridman et al., 2014). Antibiotic tolerance is a phenotypic phenomenon that transiently increases the resilience of bacterial cells during drug exposure (often at levels much higher than the MIC) prolonging cell survival during treatment (Balaban et al., 2019). Importantly, tolerance can also facilitate the evolution of AMR (Levin‐Reisman et al., 2017; Windels et al., 2019). To assess the effects on tolerance, we used a modified version of the TD test (Gefen et al., 2017), measuring the percentage of bacterial regrowth following ampicillin exposure. Cells lacking RecA or RecB demonstrated significantly reduced tolerance to ampicillin, as demonstrated by reduced regrowth of cells within the ZOI following the addition of supplementary nutrients (Figures 4a,b and A2d). Ampicillin tolerance in *recA* and *recB* mutants was restored to wild‐type levels following complementation *in trans* (Figure 4c,e). Our findings are in good agreement with previous studies, which have demonstrated that deletion of *recA* reduced the tolerance of *E. coli* to ampicillin during early exposure (Kohanski et al., 2007).

We also observed reduced tolerance for ampicillin‐resistant (Amp^R^) Δ*recA* and Δ*recB* cells (Figures 4d and A2e). These Amp^R^ strains were constructed by introducing the pWSK29 plasmid (which confers ampicillin resistance through the *bla* gene) into wild‐type and DNA repair‐deficient cells by transformation. Strains deficient in SSGR or NPS had little variation in tolerance to ampicillin in either Amp^S^ or Amp^R^ backgrounds. Our results indicate that the repair of double‐strand breaks contributes to ampicillin tolerance in both the ampicillin‐sensitive and ‐resistant backgrounds.

**Figure A3:**
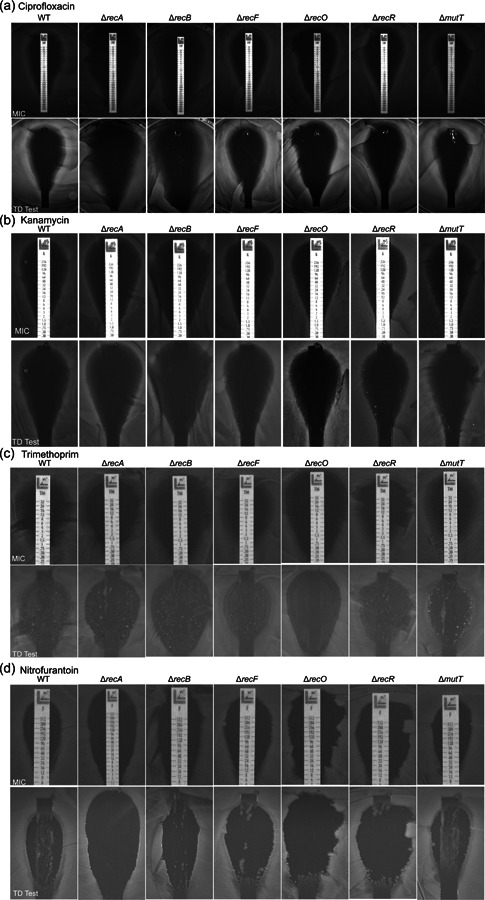
(a) Representative images of ciprofloxacin minimum inhibitory concentration (MIC) and tolerance (TD Test) plate assays for wild‐type (WT), and DNA repair‐deficient *Escherichia coli* strains. (b) Representative images of kanamycin MIC and tolerance (TD Test) plate assays for wild‐type (WT), and DNA repair‐deficient *E. coli* strains. (c) Representative images of nitrofurantoin MIC and tolerance (TD Test) plate assays for wild‐type (WT) and DNA repair‐deficient *E. coli* strains. (d) Representative images of trimethoprim MIC and tolerance (TD Test) plate assays for wild‐type (WT), and DNA repair‐deficient *E. coli* strains.

For completeness, we also examined the tolerance phenotypes of the DNA repair mutant strains following exposure to the other drugs used in this study. No tolerant cells were observed following treatment with ciprofloxacin (Figure A3a) or kanamycin (Figure A3b), this is likely due to the strong bactericidal activity of these antibiotics. Tolerant cells were observed following nitrofurantoin (Figure A3c) and trimethoprim (Figure A3d) treatment, however, no significant changes in the frequency of tolerance were observed for any DNA repair mutant. DNA repair does not appear to play a role in antibiotic tolerance to the drugs nitrofurantoin or trimethoprim.

### 3.4 DSBR defects suppress induction of the SOS response by ciprofloxacin, nitrofurantoin, and trimethoprim

**Figure 5:**
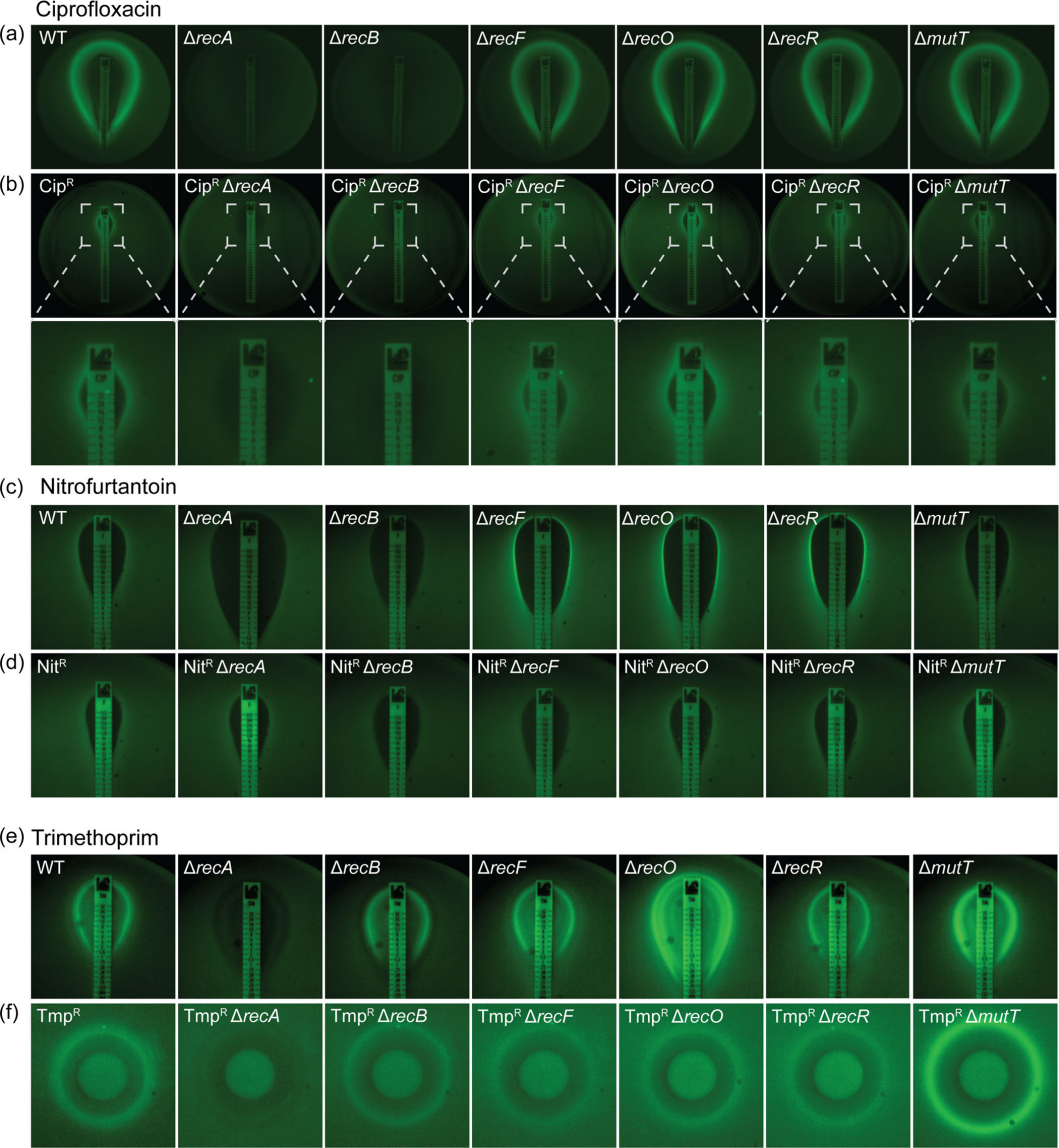
*Escherichia coli* relies on RecB for induction of the SOS response following treatment with ciprofloxacin or nitrofurantoin. (a) Expression of SOS reporter fusion P_*sulA*_‐*gfp* on a solid agar surface in ciprofloxacin‐sensitive wild‐type (WT) and DNA repair‐deficient strains grown in the presence of a ciprofloxacin MIC test strip (0.002–32 µg/ml). SOS induction is visualized as a strong fluorescence band at the border of the zone of inhibition. (b) Expression of SOS reporter fusion P_*sulA*_‐*gfp* on a solid agar surface in ciprofloxacin‐resistant (Cip^R^) wild‐type and DNA repair‐deficient strains grown in the presence of a ciprofloxacin MIC test strip (0.002–32 µg/ml). (c) Expression of SOS reporter fusion P_*sulA*_‐*gfp* on a solid agar surface in antibiotic‐sensitive wild‐type (WT) and DNA repair‐deficient strains grown in the presence of a nitrofurantoin MIC test strip (0.032–512 µg/ml). (d) Expression of SOS reporter fusion P_*sulA*_‐*gfp* on a solid agar surface in nitrofurantoin‐resistant (Nit^R^) wild‐type and DNA repair‐deficient strains grown in the presence of a nitrofurantoin MIC test strip (0.032–512 µg/ml). (e) Expression of SOS reporter fusion P_*sulA*_‐*gfp* on a solid agar surface in trimethoprim‐sensitive wild‐type and DNA repair‐deficient strains grown in the presence of a trimethoprim MIC test strip (0.002‐32 µg/ml). (f) Expression of SOS reporter fusion P_*sulA*_‐*gfp* on a solid agar surface in trimethoprim‐resistant (Tmp^R^) wild‐type (WT) and DNA repair‐deficient strains grown in the presence of a disk containing 1 mg trimethoprim.

The mutagenic SOS response is triggered by some antibiotics (Blázquez et al., 2018). We qualitatively examined the induction of the SOS response by ciprofloxacin and nitrofurantoin in DNA repair deficient cells using an agar plate‐based SOS reporter assay. SOS reporter strains were generated by transformation of DNA repair mutant cells with the plasmid pUA66‐P_sulA_‐*gfp (*Zaslaver et al., 2006 *)*, which places *gfp* under the control of the SOS‐inducible promoter P_*sulA*_. When exposed to ciprofloxacin, wild‐type, Δ*recF*, Δ*recO*, Δ*recR*, and Δ*mutT* cells exhibited robust SOS induction, manifesting as a strong fluorescence band at the border of the ZOI (Figure 5a and Movie S1: https://doi.org/10.6084/m9.figshare.20722312.v1). Ciprofloxacin‐induced SOS was abolished in the SOS‐defective Δ*recA* strain, as expected, as well as the DSBR defective Δ*recB* background. The same pattern of SOS induction, albeit at a reduced intensity and at higher drug concentrations, was also observed in the Cip^R^ background (Figure 5b). This result supports previous findings that SOS induction by quinolones is strongly *recB*‐dependent (Henrikus et al., 2020; Newmark et al., 2005), and further demonstrated this dependency in a ciprofloxacin‐resistant background.

When exposed to nitrofurantoin, wild‐type and Δ*mutT* cells containing the SOS‐reporter plasmid exhibited weak fluorescence signals (Figure 5c). Strains defective in SOS activation (Δ*recA*) or DSBR (Δ*recB*) resulted in no observable induction of the SOS response fluorescence (Figure 5c). Deletion of SSGR intermediates *recF*, *recO*, and *recR* resulted in high‐level SOS induction, as indicated by a bright fluorescence signal at the border of the ZOI and spreading outward (Figure 5c). Thus, deletion of *recA* or DSBR (*recB*) abolishes the SOS response under nitrofurantoin treatment. This would presumably reduce the capacity of these cells to undergo mutagenic repair associated with resistance formation. In contrast, cells lacking SSGR proteins exhibit increased SOS response in response to nitrofurantoin treatment and could potentially become highly mutagenic through this pathway.

When cells become resistant to nitrofurantoin there is a change in the genetics of the SOS response (Figure 5d). In the Nit^R^ background, cells lacking *recO*, *recR*, and *mutT* exhibited low‐level fluorescence, similar to that of Nit^R^ *rec* ^+^ cells. Deletion of *recA* and *recB* abolish the SOS response as consistent with the sensitive background, yet loss of the *recF* gene also abolished SOS in the Nit^R^ background. Thus, in both sensitive and resistant backgrounds, loss of *recA* and DSBR (*recB*) dampens the SOS response under nitrofurantoin treatment. SOS is dependent on *recF* in Nit^S^ cells and independent of *recF* in the Nit^R^ background.

In our hands, no obvious SOS response was detected in any strain following ampicillin treatment up to concentrations of 256 μg/ml (Figure A2d). This finding contrasts with previous studies (Blázquez et al., 2012; Thi et al., 2011) which have demonstrated ampicillin‐dependent SOS induction in *E. coli* using similar methods. We observed that kanamycin treatment did not induce a detectable SOS response in any strain analyzed (Figure A1d). In agreement with other studies (Baharoglu & Mazel, 2011; Kohanski et al., 2007; Thi et al., 2011), kanamycin treatment does not elicit a detectable SOS response in *E. coli*. Trimethoprim did induce a clear SOS response signal in both the sensitive and resistant backgrounds (Figure 5e,d). Disruption of *recA* eliminated SOS response in both cases. Deletion of *recB* reduced SOS in both cases. Disruption of SSGR did not reduce SOS, except for a *recO* deletion in the Tmp^R^ background. We note that the SOS signal was enhanced in the *recO* deletion in the sensitive background.

### 3.5 Putative DSBR inhibitors ML328 and IMP‐1700 exhibit off‐target effects

We next examined two putative DSBR inhibitors that have been reported in the literature, ML328 (Amundsen et al., 2012) and IMP‐1700 (Lim et al., 2019). In these studies, the two compounds demonstrated specific affinities to the *E. coli* RecBCD complex or the functionally related AddAB(RexAB) complexes from *Helicobacter pylori* and *Staphylococcus aureus*. Additionally, IMP‐1700 was claimed to potentiate ciprofloxacin activity, sensitizing a multi‐drug resistant *S. aureus* strain to clinically relevant levels of ciprofloxacin (Lim et al., 2019). While these two compounds show early promise, further work is required to confirm the mechanism of action as being inhibition of DSBR.

**Figure 6:**
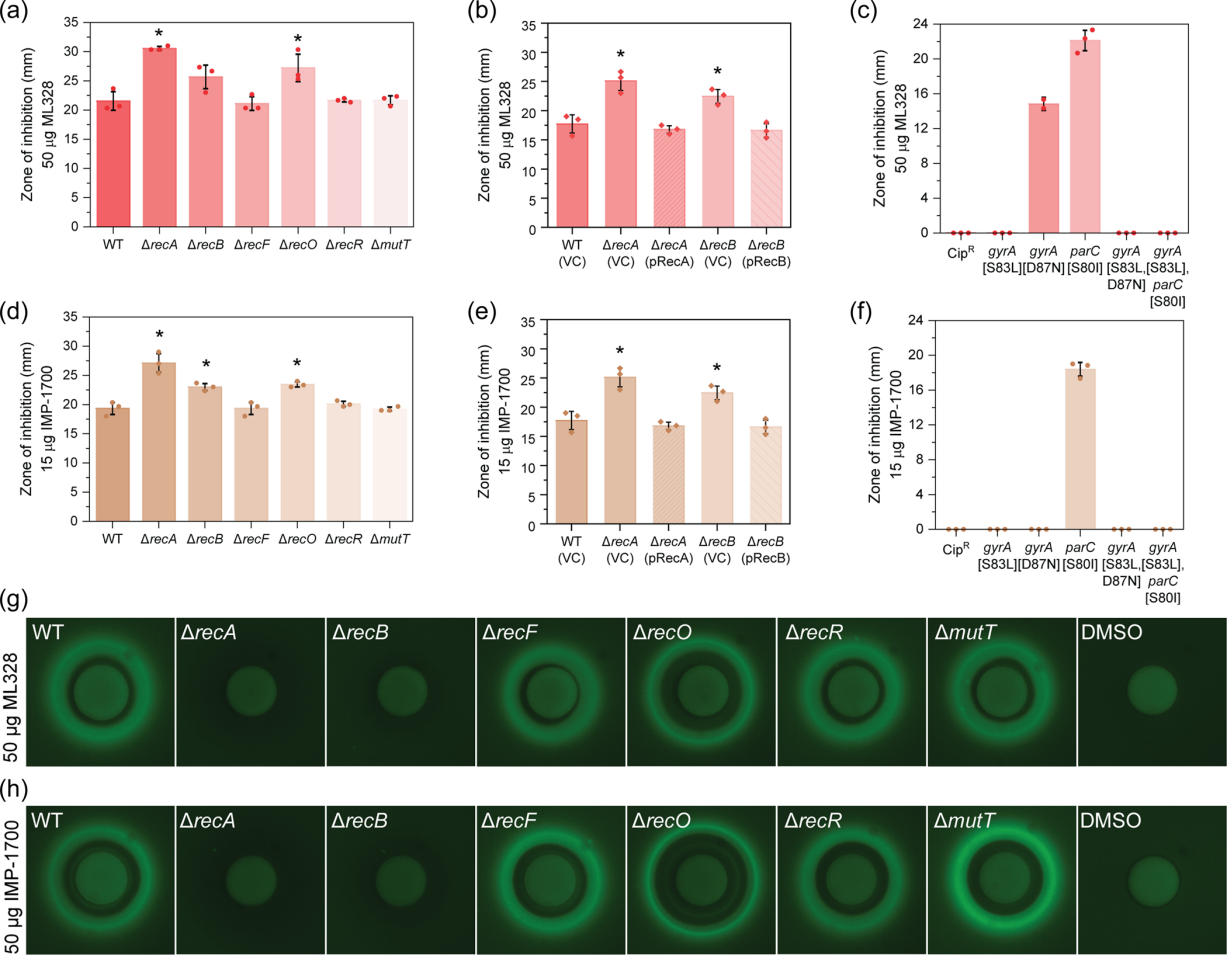
Cells lacking RecA and RecB are more sensitive than wild‐type to ML328 and IMP‐1700. (a and d) Zone of inhibition area measurements for isogenic *Escherichia coli* strains MG1655 (WT; wild‐type), Δ*recA*::Kan^R^ (HH020), Δ*recB*::Kan^R^ (EAW102), Δ*recF*::Kan^R^ (EAW629), Δ*recO*::Kan^R^ (EAW114), Δ*recR*::Kan^R^ (EAW669) and Δ*mutT*::Kan^R^ (EAW999) following disk diffusion assays with (a) 50 µg ML328 or (d) 15 µg IMP‐1700. The means and standard errors of the mean are shown based on results from at least three biological replicates. Statistical analysis was carried out using a Student's *t*‐test. An asterisk denotes statistical significance (*p* < 0.05) compared to wild‐type (WT). (b and e) Zone of inhibition area values obtained for wild‐type (MG1655) with empty vector (VC; vector control), Δ*recA* and Δ*recB* mutants with empty vector, and complemented derivatives (pRecA and pRecB, respectively) following disk diffusion assays with (b) 50 µg ML328 or (e) 15 µg IMP‐1700. The means and standard errors of the mean are shown based on results from at least three biological replicates. Statistical analysis was carried out using a Student's *t*‐test. An asterisk denotes statistical significance (*p* < 0.05) compared to wild‐type empty vector control (WT (VC)). (c and f) Zone of inhibition area values obtained for isogenic *E. coli* derivatives possessing single, double, or triple point mutations that confer ciprofloxacin resistance following disk diffusion assays with (c) 50 µg ML328 or (f) 15 µg IMP‐1700. Strains used are Cip^R^ (CH5741; *gyrA;* [S83L, D87N] and *parC;* [S80I]), *gyrA* [S83L] (LM328), *gyrA;* [D87N] (LM534), *parC;* [S80I] (LM792), *gyrA;* [S83L, D87N] (LM625) and *gyrA;* [S83L] *parC;* [S80I] (CH6179). The means and standard errors of the mean are shown based on results from at least three biological replicates. (g and h) Expression of SOS reporter fusion P_*sulA*_‐*gfp* on a solid agar surface in wild‐type (WT) and DNA repair‐deficient strains grown in the presence of (g) 50 µg ML328 or (h) 50 µg IMP‐1700. SOS induction is visualized as a strong fluorescence band at the border of the zone of inhibition.

**Figure A5:**
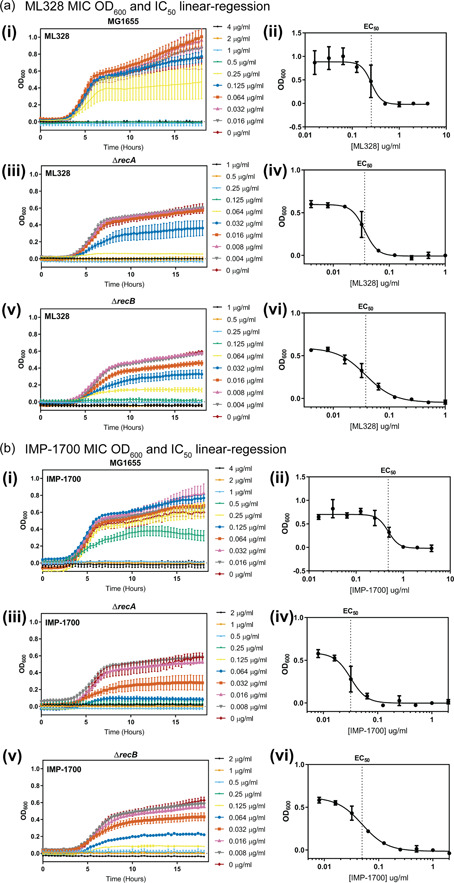
ML328 (a) and IMP‐1700 (b) OD_600_ and IC_50_ linear regression data for *Escherichia coli* strains (i and ii) MG1655 (WT; wild‐type), (iii and iv) Δ*recA*::Kan^R^ (HH020), (v and vi) Δ*recB*::Kan^R^ (EAW102). The optical density at 600 nm (OD_600_) was recorded every 20 min for 18 h. OD_600_ measurements were background corrected against no‐inoculum controls. The means and standard error of the mean are shown from three biological replicates. The minimum inhibitory concentration (MIC) was defined as the lowest concentration of compound with no growth as determined by OD_600_ readings. IC_50_ values were calculated using data from at least three biological replicates.

We examined the biological activity of these two drugs in DNA‐repair deficient *E. coli* derivatives using disc diffusion assays (Figure 6a,d). We expected that cells lacking DSBR should not show sensitivity to these compounds, since the drug target (RecB) was no longer present. However, we observed that deletion of *recB* resulted in an increased sensitivity to both drugs, suggesting that there may be off‐target effects. Cells lacking *recA* were also significantly more sensitive to both compounds. Complementation of *recA* and *recB* mutants returned sensitivity to both drugs to wild‐type levels, confirming the roles of RecA and RecB in survival following ML328 and IMP‐1700 treatment (Figure 6b,e). We quantitatively determined the MICs of ML328 and IMP‐1700 in wild‐type, Δ*recA*, and Δ*recB* mutants. Consistent with the disc diffusion data, deletion of *recA* or *recB* reduced the MICs of both compounds in *E. coli* (Table 1 and Figure A5).

**Table 1:** MIC and IC_50_ values for ML328 and IMP‐1700

Strain	ML328		IMP‐1700	
	MIC (µg/ml)	IC_50_ (µg/ml)	MIC (µg/ml)	IC_50_ (µg/ml)
WT	0.5 ± 0.00	0.255 ± 0.03	1 ± 0.00	0.481 ± 0.05
Δ*recA*	0.125 ± 0.00	0.036 ± 0.002	0.125 ± 0.00	0.032 ± 0.003
Δ*recB*	0.167 ± 0.07	0.036 ± 0.003	0.25 ± 0.00	0.049 ± 0.003

*Note*: IC_50_ values were determined by dose–response nonlinear regression. Data represent the mean and standard error based on three biological replicates.

Lim et al. (2019). showed inhibition of the SOS response in *S. aureus* following treatment with IMP‐1700. In contrast, we observed direct induction of the SOS response by these compounds in *E. coli* (Figure 6g,h). For both compounds, SOS induction was abolished in the SOS‐defective Δ*recA* strain as expected, and absent in the DSBR‐defective Δ*recB* background. No significant changes in SOS induction were observed in wild‐type, *recF, recO, recR*, or *mutT* mutants. These results suggest that induction of the SOS response in *E. coli* following treatment with ML328 and IMP‐1700 is dependent on RecB.

**Figure A6:**
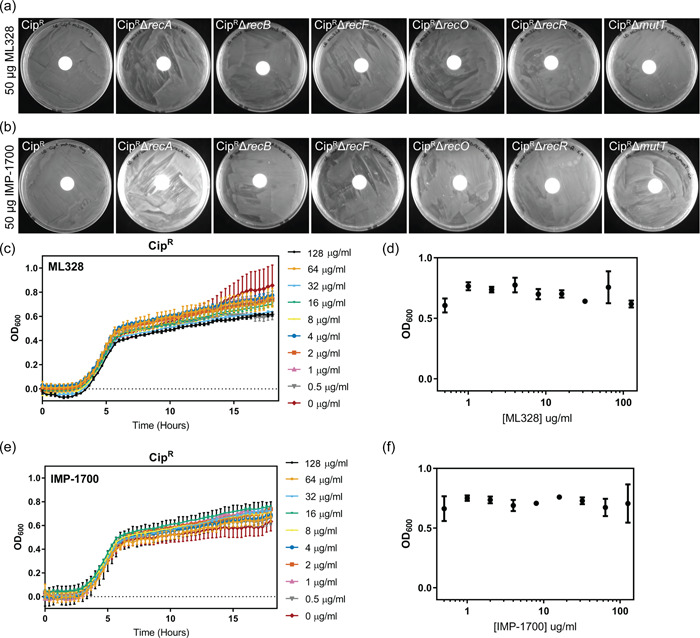
(a) and (b) Representative images of ML328 (a) and IMP‐1700 (b) disk diffusion assays obtained for isogenic ciprofloxacin‐resistant (Cip^R^) DNA repair‐deficient *Escherichia coli* strains Cip^R^ (CH5741), Cip^R^ Δ*recA* (FM002), Cip^R^ Δ*recB* (FM001), Cip^R^ Δ*recF* (FM003), Cip^R^ Δ*recO* (FM004), Cip^R^ Δ*recR* (FM005), and Cip^R^ Δ*mutT* (MV001). ML328 (c and d) and IMP‐1700 (e and f) OD_600_ and IC_50_ linear regression data for *E. coli* strains. The optical density at 600 nm (OD_600_) was recorded every 20 min for 18 h. OD_600_ measurements were background corrected against no‐inoculum controls. The means and standard error of the mean are shown from three biological replicates. IC_50_ values were calculated using data from at least three biological replicates.

Both the DSBR‐inhibiting compounds ML328 and IMP‐1700 were constructed using quinolone structural backbones. We reasoned that they each might inhibit DNA gyrase and topoisomerase IV. The authors (Lim et al., 2019) examined this potential activity using purified proteins in bulk biochemical assays and claimed no significant interactions. However, the patterns of strain sensitivity we observed with these two compounds and the strong *recB*‐dependent SOS induction mimicked the results we had obtained for ciprofloxacin. We reasoned that if ML328 and IMP‐1700 inhibited DNA gyrase and topoisomerase IV, mutations conferring resistance to quinolones may also confer resistance to these two compounds. We repeated the disc diffusion assays using Cip^R^ DNA repair deficient derivatives (Figure A6a,b). All strains examined were found to be resistant to both ML328 and IMP‐1700 at the concentrations tested. Using broth microdilution assays, the MICs of the Cip^R^ strain for both compounds were determined to be greater than 128 µg/ml (Figure A6c,d). These findings show that the point mutations *gyrA;* [S83L, D87N] and *parC;* [S80I], which typically confer resistance to quinolones, also confer resistance to ML328 and IMP‐1700. To confirm which of the three‐point mutations was most important for conferring resistance to these compounds, we repeated disc diffusion assays with isogenic *E. coli* derivatives possessing one single point mutation, or two mutations in combination (Figure 6c,f). Cells possessing a single S80I point mutation in *parC* were sensitive to both ML328 and IMP‐1700, suggesting topoisomerase is not the primary target for these two drugs. The single point mutation D87N in *gyrA* conferred resistance to IMP‐1700, but not ML328, however, the single point mutation S83L in *gyrA* conferred resistance to both compounds. These findings suggest that while these two compounds may target DSBR to some degree, in our hands the primary mode of action in *E. coli* is inhibition of gyrase and topoisomerase IV.

### 3.6 Testing of potential inhibitors of RecA and the SOS response

**Figure 7:**
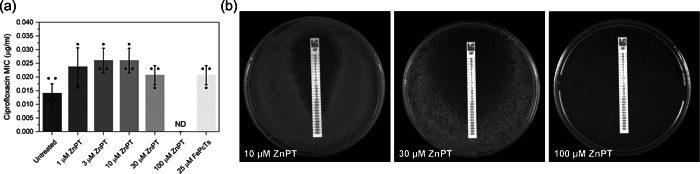
Putative RecA inhibitors show no sensitization effects in wild‐type *Escherichia coli* when compared to untreated cells. (a) Ciprofloxacin minimum inhibitory concentrations (MICs) for wild‐type *E. coli*, when left untreated or treated with a range of zinc pyrithione (ZnPT) concentrations (1, 3, 10, 30, and 100 μM) or a set concentration of 25 μM FePcTs. The means and standard errors of the mean are shown based on results from at least three biological replicates. ND means not detected. (b) Representative images of ciprofloxacin and ZnPT (at concentrations of 10, 30, and 100 μM) MIC plate assays for wild‐type cells. Images are representative plates from independent triplicate replicates.

A number of chemical compounds have previously been proposed to inhibit the SOS response via targeting RecA activity and work as resistance‐breaking compounds (Alam et al., 2016; Buberg et al., 2020; Lee & Singleton, 2004; Vareille et al., 2007). Of these, we tested two promising compounds. The first was ZnPT which has been shown to block RecA binding to single‐stranded DNA, likely preventing LexA autocleavage and subsequent SOS response induction (Bunnell et al., 2017; Crane et al., 2018). The second compound was FePcTs which has been purported to inhibit RecA ATPase activity subsequently blocking DNA strand exchange, LexA autoproteolysis, and other crucial RecA‐mediated events (Alam et al., 2016). To determine whether either of these RecA‐targeting compounds show sensitivity effects similar to a Δ*recA* mutation, a sensitivity assay using ciprofloxacin MIC test strips was undertaken. Previous work has outlined the potentiating effects of FePcTs on ciprofloxacin, with all CFUs eliminated after 24 h (Alam et al., 2016). Wild‐type *E. coli* was tested on a set concentration of FePcTs (25 μM) previously shown to have potentiating effects. In our hands, 25 μM FePcTs in conjunction with ciprofloxacin had no sensitizing effects when compared to wild‐type cells treated with ciprofloxacin alone (Figure 7a). ZnPT was also tested for synergistic sensitivity effects alongside ciprofloxacin. A range of ZnPT concentrations was tested (1, 3, 10, 30, and 100 μM). ZnPT + ciprofloxacin results showed no significant change in MIC compared to ciprofloxacin alone; except for the 100 μM ZnPT plate which had no cell growth. At 30 μM ZnPT showed a larger ZOI (Figure 7b) with single colonies outside of the ciprofloxacin diffusion range, suggesting ZnPT alone has killing effects at this concentration. These two putative RecA inhibitors did not show sensitivity effects similar to the Δ*recA* mutant.

## 4 DISCUSSION

This study demonstrated that disruption of bacterial DSBR induced sensitizing effects against multiple bactericidal antibiotics with disparate modes of action. The deletion of genes involved in DSBR (*recA* and *recB*) hypersensitized *E. coli* cells against ciprofloxacin and nitrofurantoin (Figure 1), enhanced the clearing of cells by kanamycin (Figure 2) and trimethoprim (Figure 3), and decreased tolerance to ampicillin (Figure 4). Most importantly, sensitizing effects were also observed in antibiotic‐resistant strains, raising the possibility of targeting DSBR for the development of novel resistance breakers. Additionally, activation of the highly mutagenic SOS response was found to be dependent on DSBR, raising the possibility that disrupting DSBR would limit the capacity of bacterial populations to develop further antibiotic resistance mutations. Overall, our findings establish DSBR as a promising target for the design of broad‐range resistance‐breaking compounds that could be used to dramatically enhance the effectiveness of existing bactericidal antibiotics and suppress the development of antibiotic resistance.

### 4.1 DSBR as a novel drug target

In this study, we found that disruption of DSBR induced a suite of phenotypes that could lead to more effective antibiotic treatments. Disruption of DSBR enhanced killing by five disparate classes of antibiotics. Previous studies have shown that *recA* mutants are highly sensitive to quinolones and other DNA‐damaging drugs (Machuca et al., 2021; Maeda et al., 2019; Singh et al., 2010; Thi et al., 2011). Our observations support and build upon these findings, demonstrating that *recB* mutants also share this hypersensitivity phenotype. Importantly, we observed that these sensitization phenomena also extended to drug‐resistant *E. coli* strains, suggesting for the first time that disruption of DSBR via inhibition of RecA or RecBCD might represent a viable strategy for the development of broad‐ranging antibiotic resistance breakers.

In this study, we examined *E. coli*, however, studies by others suggest that disruption of DSBR may improve the killing of other bacterial species. In *S. aureus*, DSBR pathways promote the survival of both antibiotic‐sensitive and ‐resistant bacteria following exposure to antibiotics such as fluoroquinolones, daptomycin, and nitrofurantoin (Clarke et al., 2021). In a separate study, DSBR‐deficient *Acinetobacter baumannii* were significantly sensitized to colistin, gentamycin, rifampicin, and tigecycline (Ajiboye et al., 2018). For both pathogens, the inactivation of DSBR pathways also increased susceptibility to trimethoprim and sulfamethoxazole (Aranda et al., 2011; Clarke et al., 2019). Importantly, DSBR pathways also play significant roles in the establishment and maintenance of *S. aureus* infections (Ha et al., 2020). This reliance on DSBR for infection is also true for the non‐ESKAPE pathogens *H. pylori*, *Salmonella enterica*, and *Campylobacter jejuni* (Amundsen et al., 2008; Cano et al., 2002; Gourley et al., 2017). There are a few exceptions to this finding, for example, DSBR‐deficient strains of the acid‐fast bacterium *Mycobacterium tuberculosis* do not have any infectivity defects (Heaton et al., 2014). However, this is likely due to the presence of alternate pathways that can repair double‐strand DNA breaks in these cells (Brzostek et al., 2014). Furthermore, in *Klebsiella pneumoniae*, there are interesting DSB‐induced phenotypes (Liu et al., 2020). The anticancer drug bleomycin is known to trigger DSBs. When DSBs were induced in *K. pneumoniae* either by treatment with bleomycin or by a specific CRISPR‐Cas9 catalyzed reaction, this resulted in the formation of a novel “R” biofilm. Yet when another known DSB‐inducing antibiotic, ciprofloxacin, was tested this novel biofilm was notably absent (Liu et al., 2020). These results show variable cellular response to DSBs in *K. pneumoniae*. Overall, the bulk of results, in conjunction with our own findings, suggest that DSBR inhibitors may not only potentiate antibiotic activities but also reduce the infection potential of many diverse bacterial pathogens.

At high concentrations, the aminoglycoside kanamycin and the folate inhibitor trimethoprim were more active against *recA* and *recB* mutants than against the wild‐type strain. These sensitivities were also observed in the respective resistant backgrounds. It remains unclear why the MICs of DSBR‐deficient strains were similar to wild‐type while also demonstrating notably increased sensitivities to high drug concentrations. One possibility is that high concentrations of these drugs are necessary for initiating DNA damage. Although disruption of DSBR does not significantly resensitize *E. coli* to these drugs, it does increase the efficacy of killing. It is reasonable to hypothesize that the increased killing observed here may translate to improved infection clearance times in vivo when using combinational DSBR inhibitor‐antibiotic therapeutics.

In both the sensitive and resistant backgrounds we observed a strong dependence on both RecA and RecB for tolerance following ampicillin exposure. Tolerance enables bacteria to survive exposure to high levels of antibiotics (Balaban et al., 2019). Importantly, antibiotic tolerance is a key contributor to recalcitrant infections, since these cells survive primary antibiotic treatment (Lewis & Manuse, 2019). Antibiotic tolerance can also act as a precursor for resistance development (Levin‐Reisman et al., 2017). Our discovery that ampicillin tolerance in *E. coli* relies on DSBR suggests that combinational therapy with DSBR‐inhibiting drugs may help to eliminate tolerant bacteria.

Mutagenesis is one of the major pathways through which antibiotic resistance develops in bacteria (Blázquez et al., 2018; Maslowska et al., 2019). In DNA‐damage settings, including certain antibiotic treatments, the rate of mutagenesis is elevated through activation of the SOS response (Maslowska et al., 2019). It is at concentrations of drug higher than the MIC (where often the SOS response is activated) but within a concentration range where cells are not yet effectively killed, that the development and subsequent selection for drug‐resistant mutants frequently occurs. This antibiotic concentration range is known as the mutant selection window (Drlica & Zhao, 2007). For treatments to be effective at preventing the development of mutational resistance, antibiotic concentrations need to be maintained above this mutant selection window. Alternatively, strategies could prevent mutagenesis altogether, through suppression of the SOS response. Disrupting DSBR does both. Recent findings have linked ciprofloxacin‐induced SOS activation with DSBR (Henrikus et al., 2020). Our observations are in good agreement with this study. Further, our work has established a new link between DSBR and nitrofurantoin‐induced SOS induction, since *recB* mutants did not elicit an SOS response to nitrofurantoin. Importantly, this RecB dependence for SOS induction also applies in drug‐resistant *E. coli* for both ciprofloxacin and nitrofurantoin. We hypothesize that cells unable to initiate SOS or have a reduced induction of SOS would be less likely to develop beneficial resistance mutations. Our work with LexA mutants strengthened this link as results demonstrated SOS induction‐dependent resistance phenotypes. Inhibition of SOS induction through disruption of DNA repair, specifically DSBR, may prove a promising strategy to combat the evolution of AMR.

### 4.2 Multiple bactericidal antibiotics induce double‐strand breaks

It is well characterized that ciprofloxacin‐induced DNA damage predominantly occurs in the form of double‐stranded breaks (Drlica et al., 2008, 2009; Henrikus et al., 2020). For the other antibiotics used in this study, the mechanisms that drive DNA damage remain largely unknown. Throughout this study, we observed several antibiotic‐associated phenotypes that were dependent on DSBR. Kanamycin and trimethoprim treatment at high concentrations was more effective at clearing cells lacking DSBR than wild‐type, suggesting at high drug concentrations double‐strand DNA breaks occur. We also observed reduced ampicillin tolerance in DSBR deficient strains, suggesting that long‐term exposure to ampicillin induces double‐stranded breaks. In the case of nitrofurantoin, the survival of cells following antibiotic treatment was strongly dependent on homologous recombination, including the DSBR pathway. This finding suggests that, in part, the formation of double‐stranded breaks is one mechanism by which nitrofurantoin works in *E. coli*. Taken together, this study lends further weight to the notion that DNA damage, and in particular double‐strand breaks, is a common thread between many bactericidal antibiotics.

### 4.3 New antibiotic‐induced phenotypes associated with DNA gap repair

Single‐strand DNA gaps in bacteria are commonly formed postreplication (Friedberg et al., 2005), or via exposure to DNA damaging agents, such as UV (Setlow et al., 1963) and possibly even antibiotics. If left unrepaired, these gaps can be converted into DSBs, which are highly detrimental to bacterial cells (Cox et al., 2000; Hong et al., 2017). Although it was not observed for all antibiotics tested in this study, the deletion of components involved in SSGR (*recF, recO*, and *recR*) did significantly alter some antibiotic sensitivity phenotypes. Notably, all mutants lacking components of SSGR were sensitized to nitrofurantoin, to levels equal to the DSBR‐deficient cells. This sensitization effect in SSGR‐deficient cells was also observed in the nitrofurantoin‐resistant background. This suggests that DNA damage in the form of both single‐stranded gaps and double‐strand breaks are key contributors to nitrofurantoin action in *E. coli*. One unanticipated finding related to the genetic dependencies of SOS induction in cells exposed to nitrofurantoin. MICs were significantly reduced in SSGR‐deficient strains, yet these cells demonstrated high levels of SOS relative to the wild‐type. This increased‐SOS induction behavior was not observed in Nit^R^ cells. In the case of nitrofurantoin, targeting single‐cell gap repair does increase antibiotic sensitivity, however, it may also enhance the likelihood of resistant cells forming due to elevated SOS activity.

For the drugs ciprofloxacin and trimethoprim, antibiotic‐sensitive *recO* cells were significantly sensitized while other SSGR mutants remained largely unchanged. Interestingly, the RecO protein is known to bind both single‐stranded DNA and double‐stranded DNA (Luisi‐DeLuca & Kolodner, 1994). We also saw that these RecO deficient cells exhibited an increased relative SOS signal during exposure to trimethoprim. However, for both drugs, this sensitization effect (and for trimethoprim the SOS effect) was no longer present in their respective drug‐resistant backgrounds. While further work is required to understand the basis of these phenotypes, they do lend further support to the notion that the role of RecO in SSGR may be somewhat separate from those played by RecF and RecR (Henrikus et al., 2019).

We also examined the importance of NPS in survival following antibiotic treatment. Antibiotic‐induced oxidative stress can result in the formation of highly toxic and mutagenic oxidized nucleotide bases (e.g., 8‐oxo‐dGTP) (Foti et al., 2012), which must be cleared from the nucleotide pool by MutT before they are incorporated into the genome (Fowler & Schaaper, 1997). Although MTH1 (the MutT homolog in humans) holds promise as an anticancer therapeutic target (Samaranayake et al., 2017), disruption of *mutT* does not appear to be sufficiently important for bacterial cell survival or tolerance to be clinically useful. Furthermore, bacterial cells lacking *mutT* are highly mutagenic (Fowler & Schaaper, 1997), and following trimethoprim treatment, *mutT* mutants had an increased relative induction of the mutagenic SOS response. As such, MutT would not be an appropriate target for future drug development.

### 4.4 Development of DSBR inhibitors: The challenge of off‐target effects

Since our work demonstrated multiple benefits of targeting DSBR we investigated the efficacy of the published DSBR inhibitors ML328 (Amundsen et al., 2012) and IMP‐1700 (Lim et al., 2019) in *E. coli*. Contrary to expectations, cells lacking RecB (the putative target of these compounds) were more sensitive to these two compounds than wild‐type. This result implied that there these compounds have off‐target effects in *E. coli*. Further investigation revealed that these drugs were rendered ineffective in fluoroquinolone‐resistant derivatives of *E. coli*. The putative primary target of these two drugs appears to be DNA gyrase, as a single point mutation (known to increase ciprofloxacin resistance) in *gyrA* rendered cells resistant to these compounds. In the initial studies on these compounds, bulk biochemical assays determined there was no notable inhibition of *E. coli* DNA gyrase or topoisomerase IV activity (Lim et al., 2019). However, only a single concentration of inhibitor was used to assess this inhibition activity. It is likely if the drug concentration range were extended further, gyrase inhibition may have been observed.

The Δ*recA* mutants were compared to putative inhibitors of RecA and the SOS response (ZnPT; Buberg et al., 2020) and FePcTs (Alam et al., 2016) for sensitivity effects with ciprofloxacin. Previous work has outlined the mechanism by which each of these compounds targets RecA and the subsequent SOS response, resulting in inhibition of the cell hypermutation response (Bunnell et al., 2017). In our hands, these compounds gave no significant effects when tested only for sensitization compared to a Δ*recA* mutation. Effects of 30 μM ZPT + ciprofloxacin suggest greater efficiency of killing at higher ciprofloxacin concentrations, yet no change in MIC from ciprofloxacin‐only plates. Use of ZPT at such high concentrations could be unwise as 10 μM ZPT has shown toxicity in human cells (Priestley & Brown, 1980). Twenty‐five micromolar FePcTs has previously been shown to potentiate ciprofloxacin effects, our results did now show any potentiation or sensitivity effects. While the targeting of RecA has been shown as the mechanism of action in the compounds, neither shows sensitivity effects similar to the Δ*recA* mutant. Furthermore, variable results from RecA targeting do not suggest that it is a good candidate as a drug resensitization co‐therapy.

Further work is needed to identify DSBR inhibitors with fewer off‐target effects. Nevertheless, the results of the current study highlight that disrupting bacterial DSBR produces multiple beneficial effects and the search for DSBR inhibitors is a worthwhile pursuit.

## AUTHOR CONTRIBUTIONS

**Sarah A. Revitt‐Mills**: Conceptualization (equal); investigation (equal); supervision (equal); visualization (equal); writing–original draft (equal); writing–review & editing (equal). **Elizabeth K. Wright**: Conceptualization (equal); investigation (equal); visualization (equal); writing–original draft (equal); writing–review & editing (equal). **Madaline Vereker**: Investigation (equal). **Callum O'Flaherty**: Investigation (equal). **Fairley McPherson**: Investigation (equal). **Catherine Dawson**: Investigation (equal). **Antoine M. van Oijen**: Funding acquisition (supporting); supervision (equal). **Andrew Robinson**: Conceptualization (equal); funding acquisition (lead); investigation (equal); project administration (lead); supervision (lead); writing–original draft (equal); writing–review & editing (equal).

## CONFLICT OF INTEREST

None declared.

## ETHICS STATEMENT

Protocols and procedures employed in this investigation were reviewed and approved by the institutional review committees at the University of Wollongong (GT18/05 and IBC20/02).

## Figures and Tables

**Figure A4:**
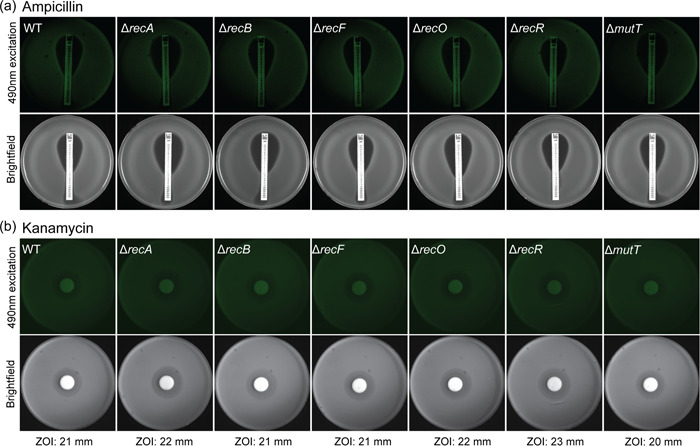
(a) Expression of SOS reporter fusion P_*sulA*_‐*gfp* on a solid agar surface in wild‐type (WT) and DNA repair‐deficient strains grown in the presence of ampicillin (0.016–256 µg/ml). Plates were visualized under 490 nm excitation (top panels) and in bright‐field (lower panel). Any SOS induction should be visualized as a strong fluorescence band at the border of the zone of inhibition. (b) Expression of SOS reporter fusion P_*recN*_‐*gfp* on a solid agar surface in wild‐type (WT) and DNA repair‐deficient strains grown in the presence of 50 µg kanamycin. Plates were visualized under a 490 nm excitation (top panels) and in bright‐field (lower panel). The zone of inhibition recorded for each strain is denoted below the bright‐field image.

## Data Availability

All data are provided in full in this paper. The sequence of plasmid pSRM3 (pRecB) is available in GenBank, accession number OP341514: https://www.ncbi.nlm.nih.gov/nuccore/OP341514. **Movie S1** is available in figshare at https://doi.org/10.6084/m9.figshare.20722312.v1 (Movie S1: Time‐lapse acquisition [5‐min intervals] of the expression of SOS reporter fusion P_*sulA*_‐*gfp* on a solid agar surface in wild‐type (WT) *E. coli* grown in the presence of a ciprofloxacin MIC test strip (0.002–32 µg/ml). SOS induction is visualized as a strong fluorescence band at the border of the zone of inhibition).

## APPENDIX 1

**Table A1:** *Escherichia coli* strains used in this study

Strain	Relevant genotype	Parent strain	Source/technique
MG1655	*recA* ^*+*^ *recB* ^*+*^ *recF* ^*+*^ *recO* ^*+*^ *recR* ^*+*^ *mutT* ^*+*^	‐	
HH020	Δ*recA*::Kan^R^	MG1655	Ghodke et al. (2019)
EAW102	Δ*recB*::Kan^R^	MG1655	Henrikus et al. (2020)
EAW629	Δ*recF*:Kan^R^	MG1655	Henrikus et al. (2019)
EAW114	Δ*recO*::Kan^R^	MG1655	Henrikus et al. (2019)
EAW669	Δ*recR*::Kan^R^	MG1655	Henrikus et al. (2019)
EAW999	Δ*mutT*::Kan^R^	MG1655	Lambda Red recombination
HH021	Δ*recA*::FRT	HH020	Ho et al. (2018)
HG356	Δ*recB*::FRT	EAW102	Henrikus et al. (2020)
MV009	Δ*recF*::FRT	EAW629	This study
SRM019	Δ*recO*::FRT	EAW114	This study
SRM020	Δ*recR*::FRT	EAW669	This study
MV005	Δ*mutT*::FRT	EAW999	This study
CH5741	Cip^R^ (*gyrA* [S83L, D87N] *parC* [S80I]) *recA* ^*+*^ *recB* ^*+*^ *recF* ^*+*^ *recO* ^*+*^ *recR* ^*+*^ *mutT*	MG1655	Huseby et al. (2017)
FM002	Cip^R^ (*gyrA* [S83L, D87N] *parC* [S80I]) Δ*recA*::Kan^R^	CH5741	Transduction of CH5741 with P1 grown on HH021
FM001	Cip^R^ (*gyrA* [S83L, D87N] *parC* [S80I]) Δ*recB*::Kan^R^	CH5741	Transduction of CH5741 with P1 grown on EAW102
FM003	Cip^R^ (*gyrA* [S83L, D87N] *parC* [S80I]) Δ*recF*::Kan^R^	CH5741	Transduction of CH5741 with P1 grown on EAW629
FM004	Cip^R^ (*gyrA* [S83L, D87N] *parC* [S80I]) Δ*recO*::Kan^R^	CH5741	Transduction of CH5741 with P1 grown on EAW114
FM005	Cip^R^ (*gyrA* [S83L, D87N] *parC* [S80I]) Δ*recR*::Kan^R^	CH5741	Transduction of CH5741 with P1 grown on EAW669
MV001	Cip^R^ (*gyrA* [S83L, D87N] *parC* [S80I]) Δ*mutT*::Kan^R^	CH5741	Transduction of CH5741 with P1 grown on EAW999
SSH021	MG1655 (pJM1071)	MG1655	Transformation of MG1655 with pJM1071
SRM009	Δ*recA*::Kan^R^ (pJM1071)	HH020	Transformation of HH020 with pJM1071
MV002	Δ*recA*::Kan^R^ (pRecA)	HH020	Transformation of EAW102 with pRecA
SRM010	Δ*recB*::Kan^R^ (pJM1071)	EAW102	Transformation of EAW102 with pJM1071
EKW008	Δ*recB*::Kan^R^ (pRecB)	EAW102	Transformation of EAW102 with pRecB
SRM011	Δ*recA*::FRT (pJM1071)	HH021	Transformation of HH021 with pJM1071
CD006	Δ*recA*::FRT (pRecA)	HH021	Transformation of HH021 with pRecA
SRM012	Δ*recB*::FRT (pJM1071)	HG356	Transformation of HG356 with pJM1071
SRM013	Δ*recB*::FRT (pRecB)	HG356	Transformation of HG356 with pRecB
COF001	Amp^R^ MG1655 (pWSK29)	MG1655	Transformation of MG1655 with pWSK29
COF002	Amp^R^ Δ*recA*::Kan^R^ (pWSK29)	HH020	Transformation of HH020 with pWSK29
COF003	Amp^R^ Δ*recB*::Kan^R^ (pWSK29)	EAW102	Transformation of EAW102 with pWSK29
COF004	Amp^R^ Δ*recF*:Kan^R^ (pWSK29)	EAW629	Transformation of EAW629 with pWSK29
COF005	Amp^R^ Δ*recO*::Kan^R^ (pWSK29)	EAW114	Transformation of EAW114 with pWSK29
COF006	Amp^R^ Δ*recR*::Kan^R^ (pWSK29)	EAW669	Transformation of EAW669 with pWSK29
COF007	Amp^R^ Δ*mutT*::Kan^R^ (pWSK29)	EAW999	Transformation of EAW999 with pWSK29
CD001	MG1655 (pUA66)	MG1655	Transformation of MG1655 with pUA66
CD002	Kan^R^ Δ*recA*::FRT (pUA66)	HH021	Transformation of HH021 with pUA66
CD003	Kan^R^ Δ*recB*::FRT (pUA66)	HG356	Transformation of HG356 with pUA66
CD004	Kan^R^ Δ*recF*::FRT (pUA66)	MV009	Transformation of MV009 with pUA66
SRM026	Kan^R^ Δ*recO*::FRT (pUA66)	SRM019	Transformation of SRM019 with pUA66
SRM027	Kan^R^ Δ*recR*::FRT (pUA66)	SRM020	Transformation of SRM020 with pUA66
CD005	Kan^R^ Δ*mutT:*:FRT (pUA66)	MV005	Transformation of MV005 with pUA66
SSH091	MG1655 (pUA66‐P_*sulA*_‐*gfp*)	MG1655	Henrikus et al. (2020)
MV003	Δ*recA*::FRT (pUA66‐P_*sulA*_‐*gfp*)	HH021	Transformation of HH021 with pUA66‐P_*sulA*_‐*gfp*
SSH111	Δ*recB*::FRT (pUA66‐P_*sulA*_‐*gfp*)	HG356	Henrikus et al. (2020)
MV011	Δ*recF*::FRT (pUA66‐P_*sulA*_‐*gfp*)	MV009	Transformation of MV009 with pUA66‐P_*sulA*_‐*gfp*
SRM028	Δ*recO*::FRT (pUA66‐P_*sulA*_‐*gfp*)	SRM019	Transformation of SRM019 with pUA66‐P_*sulA*_‐*gfp*
SRM029	Δ*recR*::FRT (pUA66‐P_*sulA*_‐*gfp*)	SRM020	Transformation of SRM020 with pUA66‐P_*sulA*_‐*gfp*
MV013	Δ*mutT*::FRT (pUA66‐P_*sulA*_‐*gfp*)	MV005	Transformation of MV005 with pUA66‐ P_*sulA*_‐*gfp*
SRM020	MG1655 (pEAW915)	MG1655	Transformation of MG1655 with pEAW915, Chen et al. (2015)
SRM037	Δ*recA*::FRT (pEAW915)	HH021	Transformation of HH021 with pEAW915
SRM021	Δ*recB*::FRT (pEAW915)	HG356	Transformation of HG356 with pEAW915
SRM022	Δ*recF*::FRT (pEAW915)	MV009	Transformation of MV009 with pEAW915
SRM023	Δ*recO*::FRT (pEAW915)	SRM019	Transformation of SRM019 with pEAW915
SRM024	Δ*recR*::FRT (pEAW915)	SRM020	Transformation of SRM020 with pEAW915
SRM025	Δ*mutT:*:FRT (pEAW915)	MV005	Transformation of MV005 with pEAW915
JW0835‐1	Δ*nfsA*::kan^R^	BW25113	Baba et al. (2006)
EKW036	Nit^R^ MG1655 Δ*nfsA*	MG1655	Transduction of MG1655 with P1 grown on JW0835‐1
EKW037	Nit^R^ Δ*recA*::FRT Δ*nfsA*	HH021	Transduction of HH021 with P1 grown on JW0835‐1
EKW038	Nit^R^ Δ*recB*::FRT Δ*nfsA*	HG356	Transduction of HG356 with P1 grown on JW0835‐1
EKW039	Nit^R^ Δ*recF*::FRT Δ*nfsA*	MV009	Transduction of MV009 with P1 grown on JW0835‐1
EKW040	Nit^R^ Δ*recO*::FRT Δ*nfsA*	SRM019	Transduction of SRM019 with P1 grown on JW0835‐1
EKW041	Nit^R^ Δ*recR*::FRT Δ*nfsA*	SRM020	Transduction of SRM020 with P1 grown on JW0835‐1
EKW042	Nit^R^ Δ*mutT*::FRT Δ*nfsA*	MV005	Transduction of MV005 with P1 grown on JW0835‐1
EKW058	Tmp^R^ MG1655 [C49765T]	MG1655	λ_RED_ recombination of MG1655 with SRP84
EKW059	Tmp^R^ Δ*recA*::Kan^R^	EKW048	Transduction of EKW048 with P1 grown on HH020
EKW060	Tmp^R^ Δ*recB*::Kan^R^	EKW048	Transduction of EKW048 with P1 grown on EAW102
EKW061	Tmp^R^ Δ*recF*::Kan^R^	EKW048	Transduction of EKW048 with P1 grown on EAW629
EKW062	Tmp^R^ Δ*recO*::Kan^R^	EKW048	Transduction of EKW048 with P1 grown on EAW114
EKW063	Tmp^R^ Δ*recR*::Kan^R^	EKW048	Transduction of EKW048 with P1 grown on EAW669
EKW064	Tmp^R^ Δ*mutT*::Kan^R^	EKW048	Transduction of EKW048 with P1 grown on EAW999
LM378	*gyrA* [S83L], *recA* ^*+*^ *recB* ^*+*^ *recF* ^*+*^ *recO* ^*+*^ *recR* ^*+*^ *mutT* ^*+*^	MG1655	Marcusson et al. (2009)
LM534	*gyrA* [D87N], *recA* ^*+*^ *recB* ^*+*^ *recF* ^*+*^ *recO* ^*+*^ *recR* ^*+*^ *mutT* ^*+*^	MG1655	Marcusson et al. (2009)
LM625	*gyrA* [S83L, D87N], *recA* ^*+*^ *recB* ^*+*^ *recF* ^*+*^ *recO* ^*+*^ *recR* ^*+*^ *mutT* ^*+*^	MG1655	Marcusson et al. (2009)
LM792	*parC* [S80I], *recA* ^*+*^ *recB* ^*+*^ *recF* ^*+*^ *recO* ^*+*^ *recR* ^*+*^ *mutT* ^*+*^	MG1655	Marcusson et al. (2009)
CH6179	*gyrA* [S83L], *parC* [S80I], *recA* ^*+*^ *recB* ^*+*^ *recF* ^*+*^ *recO* ^*+*^ *recR* ^*+*^ *mutT* ^*+*^	MG1655	Huseby et al. (2017)
EAW26	*lexA*(Def) *sulA^−^*	MG1655	Robinson et al. (2015)
RW1568	*lexA3* (Ind^−^)	MG1655	Henrikus et al. (2020); Ennis et al. (1985)

**Table A2:** Plasmids used in this study

Plasmid	Description	Source
pUA66	*Escherichia coli* low copy number vector, pSC101 *ori*, GFP reporter plasmid carrying *gfpmut2*, no promoter Kan^R^	Zaslaver et al. (2006)
pUA66‐P_*sulA*_‐*gfp*	GFP reporter plasmid carrying *gfpmut2*, *sulA* promoter, Kan^R^	Zaslaver et al. (2006)
pEAW915	pACYC184 base, p15A *ori*, GFP reporter plasmid carrying supergloGFP (from pQBI63) behind *recN* (+200 to −21) promoter, Cm^R^	Chen et al. (2015)
pWSK29	*E. coli* low copy number vector, pSC101 *ori*, Amp^R^,	Rong Fu and Kushner (1991)
pJM1071	*E. coli* base plasmid, pSC101 *ori*, Spec^R^	
pHG134 (pRecA)	RecA complementation plasmid, *recA* cloned in pJM1071 between NdeI/XbaI, Spec^R^	Ghodke et al. (2019)
pSRM3 (pRecB)	RecB complementation plasmid, *recB* plus 200 bp upstream cloned in pJM1071 between KpnI/XbaI, Spec^R^	This study. GenBank accession number: OP341514
pLH29	FLP expression plasmid, p15A *ori*, FLP recombinase under the control of *lacZ* promoter, Amp^R^	Huang et al. (1997)
pKD46	Temperature‐sensitive λ Red recombinase expression plasmid, pSC101 *ori*, rep101ts, Amp^R^	Datsenko and Wanner (2000)

**Table A3:** Oligonucleotides used in this study

Oligo	Target	Sequence (5′−3′)
SRP14	nfsA_UP_F	ggaatgatgctactggcggtg
SRP15	nfsA_DN_R	cacgcagccgcttacacg
SRP16	nfsB_UP_F	cagcagcctatgatgacggc
SRP17	nfsB_DN_R	ctggtggttgatggtctggc
SRP18	recA_DN_F	ggcgacgggatgttgattct
SRP19	recA_UP_R	cgtcaggctactgcgtatgc
SRP20	recB_DN_F	cgagggtgacggcagg
SRP21	recB_UP_R	caggcggtggtcgagcc
SRP22	recF_DN_F	gcaccgcatccagccc
SRP23	recF_UP_R	gaagcggaagagatcctcgac
SRP24	recO_DN_F	ggtaatgccgtccgctcc
SRP25	recO_UP_R	gtgaccgtggagatcgaacg
SRP26	recR_UP_F	gatcgacccgagcctgc
SRP27	recR_DN_R	cgcatcggaggcgttagag
SRP28	mutT_UP_F	gcccgtgcggttctgg
SRP29	mutT_DN_R	cgaaagcgatgactggagcg
SRP81	folA_F	cgagaacgtgccgatgtg
SRP82	folA_R	gctgctgctggggtgg
SRP83	PfolA_TOP_C>T	gactcgccagcagaatataaaattttcctcaacatcatcctcgcaccagtTgacgacggtttacgctttacgtatagtggcgacaattttttttatcggg
SRP84	PfolA_BTM_G>A	cccgataaaaaaaattgtcgccactatacgtaaagcgtaaaccgtcgtcAactggtgcgaggatgatgttgaggaaaattttatattctgctggcgagtc

## APPENDIX 2

(Figure A4)

## APPENDIX 3

ImageJ Macros to analyze percentage regrowth

**IJ1MACRO** 1: (measuring MIC plate ZOI area and area without colonies)

//step 1: duplicate image and normalize brightness and contrast

//NOTE: Renaming only works if the ROI manager is empty

//clears results table and ROI manager

run("Clear Results");

selectWindow("ROI Manager");

run("Close");

//run("Brightness/Contrast…");

run("Duplicate…", " ");

setMinAndMax(0, 4079);

//step 2: threshold the zone of inhibition using a preset auto‐threshold

setAutoThreshold("Default no‐reset");

setAutoThreshold("Mean stack");

//step 3: select the zones of inhibition and add to ROI manager

//setTool("wand");

doWand(496, 300);

roiManager("Add");

doWand(400, 300);

roiManager("Add");

roiManager("Select", newArray(0,1));

roiManager("Combine");

roiManager("Add");

roiManager("Select", 2);

roiManager("Rename", "ZOI");

//step 4: select all thresholded regions (this includes colonies in ZOI)

run("Create Selection");

roiManager("Add");

roiManager("Select", 3);

roiManager("Rename", "Colonies");

//step 5: set scale to pixels and measure zone of inhibition

run("Set Scale…", "distance = 0 known = 0 pixel = 1 unit=pixel global");

roiManager("Select", 2);

run("Measure");

//step 6: measure growth in ZOI

roiManager("Select", newArray(2,3));

roiManager("AND");

run("Measure");

**IJ1MACRO 2:** (measuring the area without colonies on the TD plate)

//step 1: duplicate image and normalize brightness and contrast

//NOTE: Renaming only works if the ROI manager is empty

//run("Brightness/Contrast…");

run("Duplicate…", " ");

setMinAndMax(0, 4079);

//step 2: threshold the zone of inhibition using a preset auto‐threshold

setAutoThreshold("Default no‐reset");

setAutoThreshold("Mean stack");

//step 3: add the original zone of inhibition to ROI manager

roiManager("Select", 2);

waitForUser("Move ROI to an appropriate position")

wait(3000)

roiManager("Add");

roiManager("Select", 4);

roiManager("Rename", "ZOI‐TD");

//step 4: select all thresholded regions (this includes colonies in ZOI)

run("Create Selection");

roiManager("Add");

roiManager("Select", 5);

roiManager("Rename", "Colonies‐TD");

//step 5: set scale to pixels and measure area of colonies in zone of inhibition

run("Set Scale…", "distance=0 known=0 pixel=1 unit=pixel global");

roiManager("Select", newArray(4,5));

roiManager("AND");

run("Measure");

//step 6: save results as csv

selectWindow("Results");

saveAs("Results")

selectWindow("ROI Manager");
